# Oncolytic viruses encoding bispecific T cell engagers: a blueprint for emerging immunovirotherapies

**DOI:** 10.1186/s13045-021-01075-5

**Published:** 2021-04-16

**Authors:** Johannes P. W. Heidbuechel, Christine E. Engeland

**Affiliations:** 1 Research Group Mechanisms of Oncolytic Immunotherapy, Clinical Cooperation Unit Virotherapy, German Cancer Research Center (DKFZ), National Center for Tumor Diseases (NCT), University Hospital Heidelberg, Heidelberg, Germany; 2 Department of Medical Oncology, University Hospital Heidelberg, Heidelberg, Germany; 3 Center for Biomedical Research and Education (ZBAF), School of Medicine, Institute of Virology and Microbiology, Faculty of Health, Witten/Herdecke University, Witten, Germany

**Keywords:** Bispecific T cell engagers, Oncolytic viruses, Cancer immunotherapy, CAR T cells, Viral vectors, Immune checkpoint blockade, Adenovirus, Vaccinia virus, Measles virus, Tumor microenvironment

## Abstract

Bispecific T cell engagers (BiTEs) are an innovative class of immunotherapeutics that redirect T cells to tumor surface antigens. While efficacious against certain hematological malignancies, limited bioavailability and severe toxicities have so far hampered broader clinical application, especially against solid tumors. Another emerging cancer immunotherapy are oncolytic viruses (OVs) which selectively infect and replicate in malignant cells, thereby mediating tumor vaccination effects. These oncotropic viruses can serve as vectors for tumor-targeted immunomodulation and synergize with other immunotherapies. In this article, we discuss the use of OVs to overcome challenges in BiTE therapy. We review the current state of the field, covering published preclinical studies as well as ongoing clinical investigations. We systematically introduce OV-BiTE vector design and characteristics as well as evidence for immune-stimulating and anti-tumor effects. Moreover, we address additional combination regimens, including CAR T cells and immune checkpoint inhibitors, and further strategies to modulate the tumor microenvironment using OV-BiTEs. The inherent complexity of these novel therapeutics highlights the importance of translational research including correlative studies in early-phase clinical trials. More broadly, OV-BiTEs can serve as a blueprint for diverse OV-based cancer immunotherapies.

## Background

### The strong rationale for combining BiTEs with oncolytic viruses

Bispecific T cell engagers (BiTEs) represent a novel class of immunotherapeutic agents. BiTEs are fusion proteins that consist of two antibody single-chain variable fragments (scFv) with one scFv binding CD3 and the second scFv binding a tumor surface antigen. BiTE binding redirects polyclonal T cells toward tumor cells independent of MHC, thereby inducing anti-tumor cytotoxicity even at low concentrations. This approach has demonstrated considerable clinical efficacy against hematological malignancies. Blinatumomab, a CD3xCD19 BiTE, has been approved for treatment of B cell malignancies (reviewed in [1]). However, due to limited serum half-life, continuous infusion is required. Systemic administration is associated with severe, potentially fatal toxicities. Efficacy against solid tumors has been limited, owing to physical barriers and an immunosuppressive tumor microenvironment (reviewed in [2, 3]). As such, BiTEs are representative for diverse emerging cancer immunotherapeutics which show remarkable efficacy in certain subgroups of patients, but limited efficacy and unacceptable toxicities for the most part [4, 5].

One strategy to overcome these limitations has gained widespread interest in the immunotherapy field: oncolytic viruses (OVs). OVs selectively infect and replicate in malignant cells, ultimately leading to tumor cell lysis. This selectivity is shared between OVs across different virus families (for a concise comparison of clinically advanced oncolytic virus platforms, see Table 1) [48]. Oncolytic virotherapy relies on tumor cell-specific changes associated with the hallmarks of cancer, including increased receptor expression, impaired antiviral response, and altered metabolism (reviewed in [49]). OV replication is thus restricted to the tumor site, leaving healthy tissue unharmed (reviewed in [50]). In addition to direct tumor debulking via lytic replication, OVs can induce stromal remodeling [51], exert anti-angiogenic effects [52, 53], and, most prominently, evoke anti-tumor immune responses (reviewed in [54, 55]). Immunogenic cell death with release of tumor-associated antigens (TAAs), danger- and pathogen-associated molecular patterns (DAMPs and PAMPs) as well as cytokines and chemokines in the course of infection promote tumor-specific immunity (reviewed in [56]). OV-mediated APC maturation and antigen (cross-)presentation can result in systemic anti-tumor immunity via priming, activation, proliferation, trafficking, memory formation, cytokine release, and cytotoxic activity of polyclonal T cells. Oncolytic virotherapy is therefore an immunotherapy in its own right and has ideal properties for combinatorial approaches (reviewed in [57–59]). The unique, multi-pronged mechanism of action circumvents the development of resistance to classical therapies. Furthermore, oncolysis can also render immune-excluded and immunosuppressed tumors sensitive to otherwise unsuccessful strategies such as immune checkpoint inhibition [60–62]. Finally, OVs can be engineered to express immunotherapeutic transgenes directly at the site of infection, i.e., the inflamed tumor, achieving high local concentrations while preventing systemic side effects (reviewed in [57]). By retargeting of OVs on the entry- or post entry-level, improved tissue specificity can be achieved to further reduce the risk of immunotherapeutic on-target, off-tumor toxicities [50]. The first FDA- and EMA-approved oncolytic virus, talimogene laherparepvec (T-VEC), a modified herpes simplex virus for the treatment of malignant melanoma, encodes GM-CSF for enhanced in situ tumor vaccination and represents the current clinical benchmark in the field (reviewed in [63]). Recent data from clinical practice indicate high rates of response to T-VEC [64]. However, mainly patients with early-stage disease benefit. Therefore, combination therapy may be required to fully exploit the potential of oncolytic virotherapy. Furthermore, anti-tumor T cell activity has been associated with favorable outcome [30, 65, 66].

**Table 1:** Selected oncolytic virus platforms in clinical development

Virus platform	Virology	Safety	Flexibility	Production	Selected candidates in clinical trials
Adenovirus [9–11]	Non-enveloped icosahedral dsDNA virus (*Adenoviridae*) can cause common cold, > 50 serotypes targeting epithelia, entry via CD46/coxsackievirus adenovirus receptor/desmoglein-2, tumor-specific via genetic modification	↓ Derived from human pathogen, can cause respiratory illness and conjunctivitis ↓ Shedding observed ↓ Viral DNA enters nucleus; theoretical concern of insertion ↑ Oncoselective when engineered, requires deregulated cell division and growth pathways (defective Rb and upregulated RAS signaling) ↑ Low concern for mutations ↑ Generally well tolerated with mild side effects	↓ Intermediate transgene capacity ↑ Helper-dependent packaging vectors available ↓ Potential for retargeting limited by structural constraints ↑ Variety of serotypes	↑ Progeny produced at high titers ↑ Purification simple due to well-defined virion size and structure	H101/Oncorine with CTx in HCC (III) [NCT03780049] DNX-2401 with ICI in GBM/GS (II) [NCT02798406] OBP-301 in melanoma [NCT03190824]; with ICI and RTx in HNSCC (II) [NCT04685499] ICOVIR-5 via carrier cells in pediatric solid tumors (I/II) [NCT01844661] [12]; with or without RTx in pediatric brain tumors (I/II) [NCT04758533] Enadenotucirev in epithelial solid tumors (I/II) [NCT02028442] [13] LOAd703 (enc. immune-stimulatory CD40L/4-1BBL) with CTx and ICI in pancreatic cancer (I/II) [NCT02705196]; with ICI in melanoma [NCT04123470] ORCA-010 in prostate cancer (I/II) [NCT04097002] CG7870 with CTx in prostate cancer (I/II) [NCT00103428]; in prostate cancer (I/II) [NCT00116155] [14] CG0070 (enc. GM-CSF) in NMIBC (II/III) [NCT01438112]; (III) [NCT04452591] ONCOS-102 (enc. GM-CSF) with CTx in mesothelioma (I/II) [NCT02879669]; with ICI in peritoneal cancer (I/II) [NCT02963831] **NG-641 (enc. FAP-TAc and CXCL9-CXCL10-IFNα) alone or with ICI and/or CTx in epithelial cancers (I) [NCT04053283]**
Coxsackievirus [15–17]	Non-enveloped icosahedral (+)ssRNA viruses (*Picornaviridae*) can cause common cold and hand-foot-and-mouth disease, > 20 serotypes, entry via ICAM-1/coxsackievirus adenovirus receptor	↓ Human pathogen ↑ Well-studied, causes mild disease ↓ Relatively high mutation rate ↓ Entry receptors widely expressed on healthy tissues ↑ Low toxicity reported (in immunocompetent adults)	↓ No genetic modification (bioselection from wild-type strains)	↑ GMP manufacturing established	CVA21/V937 in melanoma (II) [NCT01227551]; with ICI in solid tumors (I/II) [NCT04521621]
Herpes simplex virus type I [18–20]	Enveloped icosahedral dsDNA virus (*Herpesviridae*) can cause skin blisters and encephalitis, entry via heparin sulfate, nectin-1/PVRL1, and HVEM, tumor-specific via genetic modification	↓ Derived from human pathogen ↑ Modified for enhanced oncotropism and reduced neurovirulence ↑ Antiviral agent available ↓ Viral DNA enters nucleus, chromosomal integration rare but possible ↑ Good safety profile in clinical trials	↑ Multiple gene deletions and transgene insertions possible	↑ Large volume production	Talimogene Laherparepvec/T-VEC/Imlygic (enc. GM-CSF) in melanoma (II) [NCT00289016]; **(III) [NCT00769704] **[21]; with ICI in melanoma (II) [NCT04068181], with RTx in sarcoma (II) [NCT04599062] HF10 with ICI in melanoma (II) [NCT02272855]; (II) [NCT03153085] G207 in brain tumors (I/II) [NCT00028158]; with RTx in pediatric gliomas (II) [NCT04482933] RP1 with ICI in solid tumors (II) [NCT03767348]; with ICI in SCC (II) [NCT04050436]
Maraba virus [22–24]	Enveloped bullet-shaped (−)ssRNA virus (*Rhabdoviridae*), originally isolated from Brazilian sand flies, apathogenic in humans, entry via LDLR, enhanced tumor specificity via genetic modification	↑ Apathogenic in humans ↑ Well tolerated even at high doses	↑ Transgene insertion, e.g., of tumor antigens, possible via reverse genetics	↑ Rapid progeny production at high titers ↑ Efficient filtration and purification due to small size and defined shape	MG1-MAGEA3 (enc. MAGE-A3) following prime with non-replicating adenovirus (enc. MAGE-A3) in solid tumors (I/II) [NCT02285816]; with ICI in NSCLC (I/II) [NCT02879760]
Measles virus [25–29]	Enveloped pleomorphic (−)ssRNA virus (*Paramyxoviridae*), causes measles and can cause encephalitis, fusogenic, live attenuated vaccine strains, entry preferentially via CD46, also via nectin-4/PVLR4 and CD150	↑ Safety of vaccine strains well established ↑ Generally well-tolerated, different administration routes tested ↑ Natural oncotropism ↑ Very low mutation rate, no conversion to wild-type observed ↑ Replication strictly cytosolic	↑ Reverse genetics system established for convenient cloning, transgene capacity > 5 kb ↓ Structural requirements for transgene, including “rule of six” (complete genome must be divisible by six) ↑ Pre- and post-entry re -/de-targeting ↑ Envelope exchange	↓ Lower titers compared to other viruses ↓ Generation of defective interfering particles ↓ Purification challenging (pleomorphic virions, sensitive to pH and shear stress)	MV-NIS (enc. human sodium/iodide symporter) with or without CTx in multiple myeloma (I/II) [NCT00450814] [30]; in ovarian/fallopian/peritoneal cancer (II) [NCT02364713]
Parvovirus [31–34]	Non-enveloped small icosahedral rodent ssDNA virus (*Parvoviridae*), apathogenic in humans, clathrin-mediated entry via unknown receptor	↑ Apathogenic in humans	↓ Transgene capacity limited to small RNAs	↑ High-titer production ↑ Stability	H-1PV in glioblastoma (I/II) [NCT01301430] [35, 36] and in pancreatic cancer (I/II) [NCT02653313] [37]
Poliovirus [17, 38]	Non-enveloped icosahedral (+)ssRNA virus (*Picornaviridae*), can cause gastroenteritis, flu-like symptoms, and poliomyelitis, entry via CD155, live attenuated vaccine strain, enhanced oncoselectivity and reduced neurovirulence of genetically modified variants or polio-rhinovirus chimera	↓ Derived from human pathogen ↑ Abrogated neurovirulence of chimeric polio-rhinovirus ↓ Relatively high mutation rate ↓ Dose-limiting toxicities observed ↑ Well tolerated at deescalated dose ↑ Sublethal infection of APCs induces sustained immune response	↓ Small genome size restricts transgene capacity	↑ Production of polio vaccine well established	PVSRIPO in glioblastoma (II) [NCT02986178], following [39]; with ICI in glioblastoma (II) [NCT04479241]; with ICI in melanoma (II) [NCT04577807]
Reovirus [40, 41]	Non-enveloped icosahedral virus with segmented dsRNA genome (*Reoviridae*), can cause gastrointestinal and respiratory symptoms, entry via JAM-A	↓ Virus shedding observed ↓ Relatively high mutation rate ↓ Viral replication despite presence of neutralizing antibodies ↑ Well tolerated in clinical trials	↓ Genetic modification not trivial, limited transgene size ↑ Novel approaches increase engineering potential	↑ Large volume production to high titers	Reolysin in sarcoma lung metastases (II) [NCT00503295]; with CTx in HNSCC (III) [NCT01166542] Pelareorep with ICI in breast cancer (II) [NCT04445844] Wild-type Reovirus with CTx in pancreatic cancer (II) [NCT01280058]
Vaccinia virus [42–47]	Enveloped large brick-shaped dsDNA virus (*Poxviridae*), origin unclear, variants found in buffalos, cattle, and rabbits, complex structure and genome, used for vaccination against smallpox, can cause rashes and fever and rare potentially lethal complications including gangrene and neurological symptoms, multiple gene deletions enhance oncoselectivity	↓ Virus shedding observed ↓ Complications associated with vaccine ↑ Low number of high-grade adverse events reported	↑ Large genome with great potential for engineering (multiple gene deletions and insertions) ↓ Genome complexity requires careful consideration	↑ High titer production ↓ Complex purification process	Pexastimogene Devacirepvec/PexaVec/JX-594 (enc. GM-CSF) in melanoma (I/II) [NCT00429312]; with TTx in HCC (III) [NCT02562755] (terminated after interim futility analysis without safety concerns) GL-ONC1 (enc. several reporter genes) in peritoneal carcinomatosis (I/II) [NCT01443260]; with CTx in ovarian cancer (I/II) [NCT02759588] T601 (enc. a prodrug conversion enzyme) with CTx in solid tumors (I/II) [NCT04226066] TBio-6517 (enc. Flt3L, anti-CTLA4, and IL-12) with ICI in solid tumors (I/II) [NCT04301011] BT-001 (enc. anti-CTLA4 and GM-CSF) with ICI in solid tumors (I/II) [NCT04725331]

A selection of the clinically most advanced oncolytic virus platforms (see [6–8] for recent reviews) is described in the Table with a focus on their safety profile, possibilities for engineering, and scalability of production. Clinical studies found on clinicaltrials.gov (as of March 13, 2021) are listed for candidates that are at least in phase I/II and include the first registered phase I/II or higher study and the most recent or most advanced trial for each therapeutic. In addition, the phase III study leading to approval of T-VEC and the first phase I study assessing a BiTE-encoding OV are highlighted. Direct comparison of different platforms is extremely challenging; mechanisms of action are highly complex and efficacy strongly depends on tumor diseases or experimental models. For more detailed information on specific viruses, the interested reader is referred to indicated reviews. Further relevant considerations for OV therapy include the route of administration and pre-existing and induced anti-viral immunity (reviewed in [7])

ds, double-stranded; (−), negative-sense; (+), positive-sense; ss, single-stranded; ICD, immunogenic cell death; CTx, chemotherapy; RTx, radiotherapy; ICI, immune checkpoint inhibition; ITx, other immunotherapy; TTx, targeted therapy (small molecule; enzyme inhibitor); I/II/III, clinical trial phase; HCC, hepatocellular carcinoma; GBM, glioblastoma multiforme; GS, gliosarcoma; HNSCC, head and neck squamous cell carcinoma; enc., encoding; GM-CSF, granulocyte macrophage colony-stimulating factor; NMIBC, non-muscle invasive bladder cancer; ICAM, intercellular adhesion molecule; GMP, good manufacturing practice; PVRL, poliovirus-receptor-like; HVEM, herpes virus entry mediator; LDLR, low density lipoprotein receptor; SCC, squamous cell carcinoma; MAGE, melanoma antigen; NSCLC, non-small cell lung cancer; NIS, human sodium/iodide symporter; H-1PV, H-1 protoparvovirus; APCs, antigen-presenting cells; JAM-A, junctional adhesion molecule A; CTLA4, cytotoxic T lymphocyte-associated protein 4; Flt3L, Flt3 ligand; IL12, interleukin-12

**Fig. 1:**
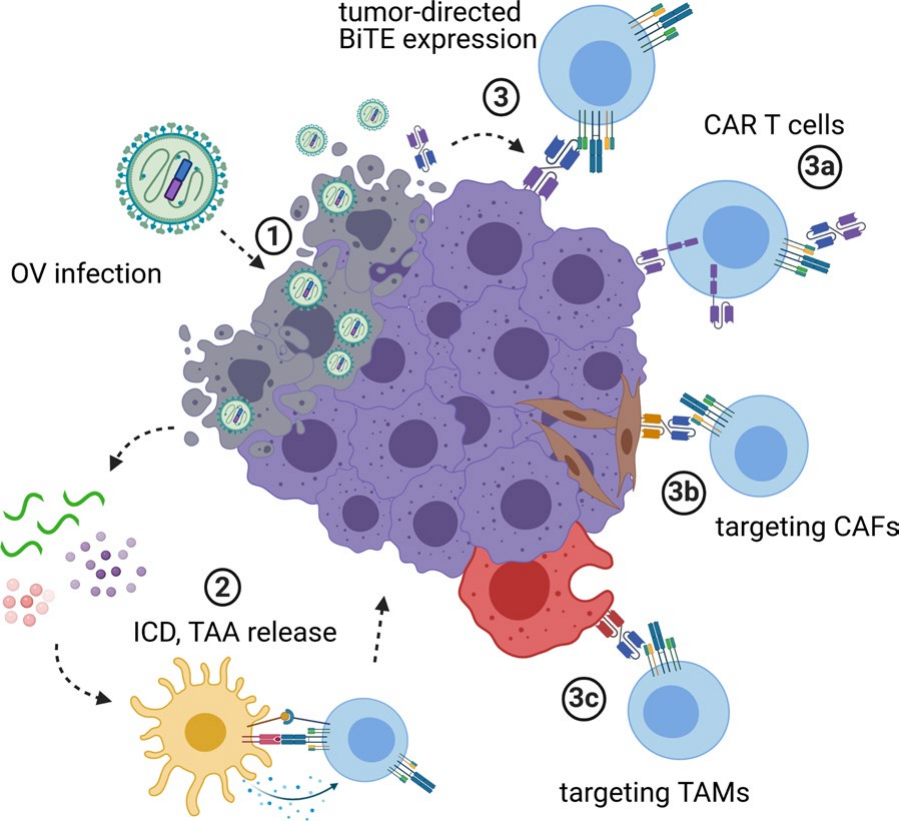
BiTE-encoding oncolytic viruses for cancer immunotherapy. Oncolytic viruses (OVs) selectively infect tumor cells, followed by lytic replication (**1**). In addition to direct tumor debulking, viral oncolysis triggers the release of danger- and pathogen-associated molecular patterns, cytokines, chemokines, and tumor-associated antigens (**2**). Upon immunogenic tumor cell death (ICD), local inflammation as well as innate and adaptive anti-tumor immune responses can set the stage for effective immunotherapy. Bispecific T cell engagers (BiTEs) redirect T cells to tumor cell surface antigens. OVs can be engineered for tumor-directed BiTE expression to benefit from high BiTE concentrations at the inflamed tumor site, while avoiding systemic toxicities (**3**). Preclinical studies have shown efficacy of this approach, utilizing BiTE-encoding OVs to engage endogenous or adoptively transferred T cells, including genetically modified CAR T cells (**3a**). Aside from direct tumor cell targeting, OV-BiTEs can also be used effectively to target immunosuppressive cells of the tumor microenvironment such as cancer-associated fibroblasts (**3b**) and tumor-associated macrophages (**3c**). Created with BioRender.com

Accordingly, the combination of BiTEs and OVs promises mutual benefits (see schematic in Fig. 1): OV infection induces local inflammation and attracts T cells to tumors, which can be redirected to tumor cells by BiTEs. In addition, encoding BiTEs in OVs can overcome BiTE limitations. This mode of delivery can maximize local concentrations at the tumor site and support penetration into solid tumors while reducing systemic exposure, thereby increasing the therapeutic window.

Efficacy of the OV-BiTE approach, especially against solid tumors, has been postulated by several groups [67–69] and investigated by a collection of recent preclinical studies. Herein, we review these approaches, focusing on BiTE target selection, transgene design, virus and BiTE characterization in vitro as well as the models used to analyze efficacy of the different BiTE-encoding oncolytic viruses (OV-BiTEs). We first address original research articles about OV-BiTEs, followed by publications featuring OV-BiTE combinations with additional immunotherapeutics. Lastly, we cover literature on OV-encoded BiTEs that target tumor-promoting cell types of the microenvironment rather than tumor cells directly. While providing an overview on the existing literature and highlighting the contributions of each publication to the current state of the field, we aim at deducing the most important open questions to be addressed for successful clinical implementation. This review article specifically discusses the existing literature on OVs encoding BiTEs. However, more broadly, OV-BiTEs are an example for the benefit of combining OVs with other immunomodulation strategies for safe and effective cancer immunotherapy. Along these lines, OV-BiTE can serve as a blueprint for development of advanced OV immunotherapeutics.

### Preclinical study design

**Fig. 2:**
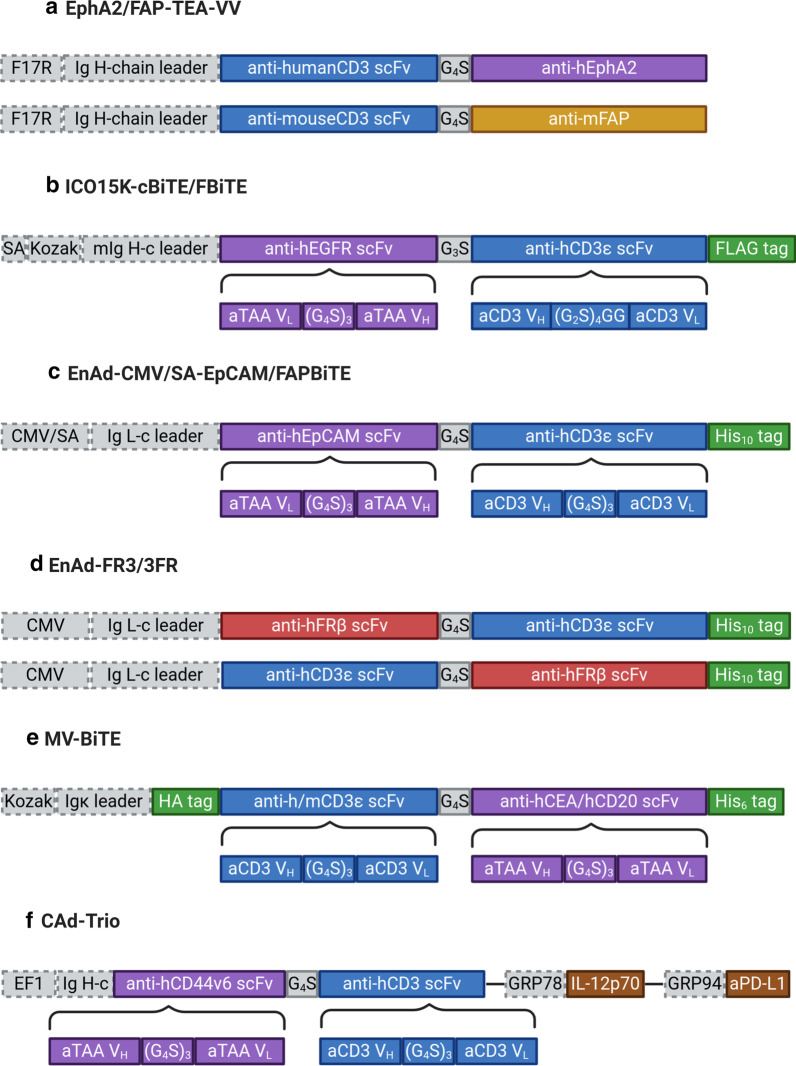
Oncolytic virus transgene cassettes encoding bispecific T cell engagers. Generally, BiTE sequences comprise single-chain variable fragments (scFvs) targeting CD3 (blue) and either a tumor-associated antigen (TAA, purple) or cell surface antigens expressed on cancer-associated fibroblasts (yellow) or tumor-associated macrophages (red). Variable heavy (V_H_) and light (V_L_) chains of scFvs are connected by flexible, non-immunogenic glycine-serine (G/S) linkers. Most constructs harbor peptide tags for detection and/or purification purposes (green). Transgenes are preceded by regulatory domains including promoters (F17R, SA, CMV, EF1, GRP78, GRP94), a Kozak sequence for efficient translation, and leader sequences coding for secretory signaling peptides derived from immunoglobulins (all in grey). **a** BiTEs specific for human Ephrin type 2 receptor (EphA2) [70] and murine fibroblast activation protein (FAP) [101], respectively, are encoded by oncolytic Vaccinia viruses (VV). **b** ICOVIR-15-derived adenoviral vectors have been engineered to encode BiTEs targeting human epithelial growth factor receptor (EGFR) (cBiTE) [73, 74, 86] or FAP (FBiTE, not shown) [107]. **c** Enadenotucirev (EnAd)-derived adenoviral vectors encode BiTEs targeting human epithelial cell adhesion molecule (EpCAM) [77], FAP (not shown) [106], or *B. pertussis* filamentous hemagglutinin adhesin (FHA, not shown) as a control, under control of either the constitutive cytomegalovirus (CMV) promoter or the adenoviral major late promoter via a splice acceptor (SA) site. **d** EnAd has also been engineered to express BiTEs specific for human folate receptor β (FRβ) or FHA (control, not shown), arranged in different orders with the CD3-targeting moiety being either C- or N-terminally [108]. **e** Four different BiTE transgene cassettes for oncolytic measles viruses (MV) have been designed, specific for either human or murine CD3 and either human carcinoembryonic antigen (CEA) or CD20 [82]. **f** Employing a combinatorial adenoviral vector system (CAd) with a replication-competent oncolytic adenovirus (not shown) and a helper-dependent vector, three immunomodulators have been encoded *in cis*; a BiTE targeting human CD44v6, a single-peptide interleukin-12 (IL-12p70), and an inhibitor of programmed death-ligand 1 (aPD-L1) [94]. TEA, T cell engager; F17R, late Vaccinia promoter; Ig, immunoglobulin; H-c, heavy chain; L-c, light chain; h, human; m, mouse; SA, splice acceptor for adenoviral major late promoter; CMV, cytomegalovirus promoter; HA tag, peptide from influenza A hemagglutinin; EF1, constitutive EF-1 α promoter; GRP78/94, commercial hamster and human promoters, respectively. Created with BioRender.com

In general, the publications featured in this review follow a similar structure. This study design is typical for preclinical investigations of immunomodulatory OVs. First, the design of BiTE-encoding vectors is described, and we will highlight the main characteristics of the transgene sequences and expression cassettes in this review (see Fig. 2 for a comparative illustration). Subsequently, the viral vectors and transgene products are characterized. Viral replication kinetics and direct tumor cell killing are assessed by progeny quantification and metabolic-, impedance-, or flow cytometry-based cell viability assays, respectively. BiTE expression and secretion are shown via SDS-PAGE and immunoblotting of cell-free supernatant of virus-infected cells. Binding specificity of BiTEs to their cognate antigens and antigen-expressing cells is assessed by ELISA and/or flow cytometry assays. Importantly, BiTE functionality is investigated using in vitro co-culture assays with target cells and immune effector cells including appropriate controls. Although establishing the basic feasibility of encoding functional BiTEs by oncolytic viruses is not trivial, this review focuses mainly on efficacy analyses performed in ex vivo and in vivo models of cancer immunotherapy with OV-BiTEs, comparing the different models and pointing out the benefits and limitations of each approach (see Table 2 for a concise summary).

**Table 2:** Preclinical studies investigating oncolytic viruses encoding bispecific T cell engagers

Study	(1) Oncolytic virus			(2) Immune effects	(3) Anti-tumor effects
	Vector platform	BiTE targets	Highlights		
Yu et al. (2014)	Oncolytic Vaccinia virus (VV), derived from Western Reserve vaccine strain	EphA2	First OV-BiTE agent described in the literature	In vitro: T cell effector cytokine production and cytotoxicity In vivo: T cell effector cytokine production T cell proliferation requires exogenous IL-2	SCID mice with A549 xenografts s.c. tumors pre-mixed with PBMCs, virus i.p.: no tumor engraftment i.v. lung colonization model, PBMC and virus mix i.v.: delayed tumor progression and prolonged survival
Fajardo et al. (2017)	Oncolytic adenovirus (AdV), derived from ICOVIR-15 K	EGFR		In vitro*:* T cell activation, proliferation, cytotoxicity, effector, and proinflammatory cytokine production In vivo: transient increase in intratumoral T cell abundance (HCT116 model) no T cell-mediated depletion of virus	SCID/beige mice with s.c. xenografts A549 tumors, virus i.t., PBMCs i.v.: delayed tumor progression HCT116 tumors, virus i.v., pre-activated T cells i.v., IL-2 i.p.: reduced tumor growth
Barlabé et al. (2019)	AdV (Fajardo et al. 2017)	EGFR	OV delivery via menstrual blood-derived mesenchymal stem cells (MenSCs)	In vitro: T cell cytotoxicity In vivo: reduced viral load vs. unmodified virus	NSG mice with s.c. A549 xenografts i.v. PBMCs, i.p. virus/virus-infected MenSCs: delayed tumor growth vs. OV-BiTE application without MenSCs/MenSCs carrying unmodified virus
Freedman et al. (2017)	AdV, derived from enadenotucirev (EnAd)	EpCAM, FHA (control)	First OV-BiTE study to include efficacy studies in primary, patient-derived model systems	In vitro: CD4^+^ and CD8^+^ T cell activation, proliferation, effector and inflammatory cytokine production, degranulation, cytotoxicity (recombinant BiTE from transfected cells); T cell activation and cytotoxicity via apoptosis induction (OV-BiTE) Ex vivo: T cell activation, proliferation, degranulation, cytotoxicity	Tumor cell depletion in ex vivo malignant peritoneal ascites and pleural effusions containing tumor cells, immune cells, stromal cells, and soluble immunosuppressive factors
Speck et al. (2018)	Oncolytic measles viruses, derived from Edmonston B vaccine strain	CEA, CD20	BiTEs engineered to target human and murine CD3ε, respectively, for use in complementary mouse models and as controls; first study to show superiority of OV-BiTE to purified BiTE	In vitro: T cell cytotoxicity, effector and inflammatory cytokine production In vivo: no negative selection of BiTE target antigen, no BiTE detected in serum following i.t. injection (PDX model); increased intratumoral mT cell levels and effector-to-regulatory T cell ratio; increased expression of T cell activation, differentiation, proliferation, and exhaustion markers (B16 model)	NSG mice with s.c. patient-derived xenografts, PBMCs i.t., virus i.t.: delayed tumor progression and prolonged survival C57BL/6J mice with s.c. MC38/B16 tumors expressing human antigens, endogenous mT cells, virus i.t.: Delayed tumor progression, prolonged survival, long-term remissions with immune protection; efficacious also in MV-immune animals; no significant difference in efficacy compared to UV-inactivated, i.e., non-replicative, virus
Wing et al. (2018)	AdV (Fajardo et al. 2017)	EGFR	First study describing combination of OV-BiTE with CAR T cells	In vitro: CAR T cell cytotoxicity toward BiTE-targeted tumor cells, T cell activation, effector cytokine production and proliferation In vivo: increased intratumoral abundance of CAR T cells, CAR T cell activation and proliferation (Panc-1 model)	NSG mice with s.c. xenografts, virus i.t., FRα-CAR T cells i.v HCT116 (CAR target high) tumors: delayed tumor growth, prolonged survival Panc-1 (CAR target low): delayed tumor growth
Porter et al. (2020)	AdV plus helper-dependent adenovirus encoding immunomodulators	CD44v6, CD19 (control)	Additional transgenes IL-12p70, PD-L1 inhibitor	In vitro: T cell activation, differentiation (T_H_1), exhaustion In vivo: CAR T cell activation, lower CAR levels at the tumor site	NSG mice with xenografts FaDu/CAPAN-1 tumors s.c., virus i.t., HER2-/PSCA-CAR T cells i.v.: Similar efficacy for immunomodulatory vectors with and without BiTE transgene Orthotopic FaDu/FaDu-HER2^−/−^ xenografts, virus i.t., HER2-CAR T cells: Trends toward reduced tumor load and prolonged survival
Yu et al. (2017)	VV	FAP	BiTE targeting CAFs instead of tumor cells; first study describing TME targeting via BiTE-encoding OV	In vitro: T cell effector cytokine production, cytotoxicity In vivo: Increased intratumoral T cell infiltration, effector cytokine production, B16-specific T cell responses (ELISpot)	C57BL/6J mice with B16 tumors s.c. model with virus i.t. and uninjected contralateral tumors: Correlation of FAP^+^ cell depletion with increased viral load in injected tumors; delayed tumor progression i.v. B16F10 lung colonization model with virus i.v.: Reduced number of tumor nodules
Freedman et al. (2018)	EnAd AdV	FAP	CAF-targeting BiTE	In vitro: CD4^+^ and CD8^+^ T cell activation, degranulation and cytotoxicity, T cell proliferation, effector cytokine production (recombinant BiTE from transfected cells), T cell activation and cytotoxicity via induction of apoptosis (OV-BiTE) Ex vivo: T cell activation, effector cytokine production, proliferation, cytotoxicity, reduction in TGF-β levels, differential gene expression—upregulation of T cell-associated genes and chemokines and antigen-presenting machinery, downregulation of fibroblast-associated genes and chemokines, and shift from M2 to M1 macrophage markers (malignant exudates), T cell activation, effector cytokine production, cytotoxicity via induction of apoptosis (prostate tumor biopsies)	Ex vivo malignant peritoneal ascites and pleural effusions: Reduction in FAP^+^ cells Ex vivo thin tissue slices from prostate cancer samples: Stromal degradation
Sostoa et al. (2019)	ICOVIR-15K AdV	FAP	CAF-targeting BiTE; recognizes both human and mouse FAP	In vitro: CD4^+^ and CD8^+^ T cell proliferation, T cell activation, effector cytokine production, cytotoxicity In vivo: Increased intratumoral T cell accumulation (A549 model)	NSG mice with s.c. A549/HPAC xenografts, virus i.t., T cells i.v.: FAP depletion, delayed tumor progression, prolonged survival
Scott et al. (2019)	EnAd AdV	FRβ, FHA (control)	BiTE targeting TAMs instead of tumor cells; comparison of different scFv orders; study also reports on trispecific T cell engagers	In vitro: T cell activation and cytotoxicity, also in presence of ascites fluid (recombinant BiTE from transfected cells) Ex vivo: CD4^+^ and CD8^+^ T cell activation and proliferation, T cell effector cytokine production and cytotoxicity (for both recombinant BiTE from transfected cells and OV-BiTE)	CD11b^+^ CD64^+^ target cell reduction in ex vivo malignant peritoneal ascites and pleural effusions containing tumor cells, immune cells, stromal cells, and soluble immunosuppressive factors

FHA, filamentous hemagglutinin adhesin (*B. pertussis*)

### First studies providing proof-of-principle for the OV-BiTE concept

In 2014, Yu et al. published the first study of a T cell engager-armed oncolytic virus. This study introduced an oncolytic Vaccinia virus (VV) encoding an EphA2-targeted T cell engager (EphA2-TEA-VV) [70]. The BiTE sequence featured scFvs targeting the human CD3-ε chain and an EphA2 epitope. This epitope is accessible on malignantly transformed but not on healthy epithelial cells [71]. The construct was expressed under control of a late promoter (F17R) to facilitate efficient viral replication. Yu et al. analyzed EphA2-TEA-VV using the EphA2-positive human lung cancer cell line A549 as target cells and human peripheral blood mononuclear cells (PBMCs) and PBMC-derived T cells, respectively, as effector cells. Virus characteristics and transgene functionality were tested in vitro. In vivo studies were performed in immunocompromised SCID/beige mice. A549 tumor cells were pre-mixed with PBMCs and injected subcutaneously (s.c.). EphA2-TEA-VV was injected intraperitoneally (i.p.) immediately thereafter. This treatment prevented tumor development completely, as opposed to treatments with PBS or control virus encoding GFP. These effects were associated with upregulation of effector cytokines. In an intravenous (i.v.) A549 lung colonization model, i.v. co-injection of PBMCs with EphA2-TEA-VV one week after tumor challenge significantly delayed tumor progression compared to control virus and monotherapies.

This study was the first to demonstrate functionality of OV-encoded BiTEs. However, the approach remained to be tested in models closer to clinical reality, where a lack of intratumoral T cell infiltration, an immunosuppressive microenvironment, and heterogenous target antigen expression are frequently observed [72].

Three years after this initial study, work focusing on an oncolytic adenovirus (AdV) encoding an EGFR-targeting BiTE (ICO15K-cBiTE) was published by Fajardo et al. [73]. The scFv targeting epidermal growth factor receptor (EGFR) was derived from cetuximab, a monoclonal antibody approved for treatment of metastatic colorectal cancer. The transgene was placed under control of a late promoter as in Yu et al., in this case the adenoviral major late promoter. In vivo efficacy of ICO15K-cBiTE was tested in immunocompromised SCID/beige mice bearing established s.c. HCT116 or A549 tumors. In the HCT116 human colorectal cancer model, pre-activated PBMC-derived T cells were injected i.v. and IL-2 was administered i.p. to support T cell viability and function. In this model, intratumoral (i.t.) treatment with ICO15K-cBiTE, but not with PBS or control virus, led to accumulation of T cells in 50% of tumors on days four to eight post injection. Both viral and cBiTE mRNA were detected in tumor tissue following i.t. virus injection and systemic T cell administration. In another experiment, i.v. treatment with ICO15K-cBiTE followed by three courses of T cell transfer plus IL-2 i.p. resulted in delayed tumor growth compared to groups receiving parental virus or PBS. In the A549 model, the combination of i.t. ICO15K-cBiTE with two courses of unstimulated PBMCs without IL-2 support significantly delayed tumor growth compared to control groups receiving parental virus or PBS or the respective monotherapies without PBMCs. Immunohistochemical analyses showed equal abundance of viral proteins in all virus-treated groups irrespective of PBMC administration, indicating virus persistence at the tumor site despite the presence of effector T cells.

Taken together, this study confirmed the functionality of BiTE-encoding OVs previously shown by Yu et al. using an alternative OV platform and a distinct target antigen in two xenograft models with transfer of either PBMCs or pre-activated T cells as effectors. Regarding the OV platform choice, the authors speculate that AdV might exhibit increased tumor cell specificity, but a reduced oncolytic rate and immunogenicity compared to VV. Compared to the previous study, the tumors had been established for a longer time before treatment and effector cells were not co-injected either with tumor cells or virus to ensure more realistic conditions. Furthermore, induction of T cell proliferation without exogenous IL-2 and prolonged intratumoral detection of viral transcripts and proteins, unimpaired by T cell activity, supported the notion of ongoing oncolysis, BiTE production, and T cell redirection in situ.

To increase systemic availability of ICO15K-cBiTE, Barlabé et al. utilized mesenchymal stem cells derived from menstrual blood (MenSCs) as carriers in a follow-up study [74]. The concept of AdV delivery via carrier cells was already investigated in clinical trials (NCT01844661, NCT04758533) [12]. A preclinical attempt with an oncolytic adenoviral vector not encoding an immunotherapeutic transgene had shown MenSC-mediated tumor delivery of the virus and modest anti-tumor efficacy [75, 76]. Barlabé et al. applied this approach in NSG mice bearing s.c. A549 tumors that had received human PBMCs i.v.. In this model, only i.p. application of MenSCs infected with ICO15K-cBiTE significantly delayed tumor growth, in contrast to PBS, ICO15K alone, ICO15K-cBiTE alone, and ICO15K-infected MenSCs, respectively. High intratumoral BiTE transgene expression was confirmed via RT-qPCR but correlated with reduced intratumoral adenovirus copies. The authors attributed this to competition between BiTE transgene and viral genes. T cell-mediated antiviral effects could provide an alternative explanation. Taken together, this study demonstrated tumor-targeted delivery of OV-BiTEs via carrier cells and anti-tumor efficacy of the strategy, however only in an immunodeficient mouse model lacking key components of the antiviral immune response such as neutralizing antibodies.

Thus, efficacy of the OV-BiTE approach still remained to be demonstrated in models with a more relevant immune contexture. This shortcoming was addressed by Freedman et al., who studied AdVs encoding an EpCAM-targeting BiTE in patient-derived ex vivo models. In this study, BiTEs were placed either under control of the constitutive CMV promoter (EnAd-CMV-EpCAMBiTE) or preceded by a splice acceptor (SA) site of the major late promoter (EnAd-SA-EpCAMBiTE), the latter restricting BiTE expression to cells supporting productive AdV infection [77]. Similar to EGFR, epithelial cell adhesion molecule (EpCAM) is frequently overexpressed on carcinomas but also present in healthy tissues, hindering systemic targeting due to toxicities (reviewed in [78]).

Importantly, the authors demonstrated T cell-mediated anti-tumor efficacy of the BiTE-encoding adenoviral vectors using tumor-immune cell mixtures from patient-derived pleural effusions and ascites fluids as described in [79]. This study was the first to show OV-BiTE efficacy in primary samples comprising matched tumor cells and T cells, immunosuppressive factors such as IL-10, and an exhausted effector T cell phenotype. Results were comparable between samples from several individual patients with distinct underlying malignancies. For EnAd-SA-EpCAMBiTE, transgene expression to was restricted to EpCAM-positive tumor cells in contrast to EnAd-CMV-EpCAMBiTE, which induced BiTE expression also in macrophages and other cells present in the malignant fluids.

This article described a second BiTE-armed AdV with a different target antigen, developed independently of ICO15K-cBiTE. Efficacy was demonstrated in patient-derived exudates as models for tumor-immune cell interactions. Successful application in these models highlights the potential of OV-directed BiTE expression under immunosuppressive conditions. In addition, the number of EpCAM-negative stromal cells present in the malignant fluids seemed to be unaffected by BiTE- or virus treatment, indicating specificity and safety of the approach. One obvious drawback of such models is the lack of three-dimensional structures and the inability to investigate the biodistribution of agents and cells. However, bloodstream stability and applicability of systemic administration of EnAd had been demonstrated in clinical trials [80, 81]. Nevertheless, the well-mixed, liquid systems applied in this study failed to show superiority of the strategy to encode the BiTE on the OV backbone. In these experimental systems, purified BiTE led to similar or sometimes superior efficacy compared to the BiTE-encoding viruses, which may be attributed to higher overall BiTE concentrations. A potential benefit of OV-BiTE, compared to direct application of the BiTE itself, thus remained to be demonstrated.

This was achieved in a 2018 study reporting on oncolytic measles viruses (MV) encoding BiTEs targeting the human model tumor antigens CEA and CD20, respectively [82]. The transgene cassette was inserted downstream of the measles virus hemagglutinin open reading frame to balance viral replication capacity and transgene expression. Viruses encoding BiTE variants containing scFvs targeting either human or murine CD3 were generated as described in [83] in order to investigate MV-BiTE in both patient-derived xenograft and immunocompetent mouse models.

Patient-derived xenografts were established by s.c. injection of colorectal carcinoma cultures in immunodeficient NSG mice. Mice receiving one i.t. injection of PBMCs followed by MV-CEA-BiTE i.t. on four consecutive days survived significantly longer than animals receiving either of the monotherapies. In contrast to i.v. administration, BiTE serum levels remained below the detection limit following i.t. injections of the virus. Both BiTE and viral N protein expression levels in tumor tissue remained stable over ten days post treatment. However, long-term survival was not observed, which was associated with a lack of T cell persistence, but not with MV-BiTE clearance or BiTE target antigen loss. Importantly, a fully immunocompetent mouse model was also employed. C57BL/6 mice were s.c. engrafted with tumors derived from the syngeneic murine melanoma cell line B16, expressing the human antigens CD46 and CD20 for MV entry and as a BiTE target, respectively (B16-CD20-CD46). In this model i.t. MV-CD20-BiTE treatment significantly prolonged survival compared to unmodified virus and virus encoding a non-targeting control BiTE. This correlated with increased intratumoral T cell infiltration and an enhanced T cell effector phenotype as evidenced by flow cytometry and targeted transcriptome analyses. Importantly, long-term survivors were protected from re-challenge with parental B16 cells, indicating activation of endogenous T cells specific for B16 melanoma antigens as opposed to human CD20 or CD46 antigens. This suggests antigen spread, potentially induced by viral oncolysis and supported by BiTE-mediated T cell redirection and recruitment. Interestingly, not only T cell activation, differentiation and proliferation genes were upregulated, but also genes associated with T cell inhibition and exhaustion. This provides a rationale for additional combinations with immune checkpoint inhibitors, which may further increase treatment efficacy. In the B16-CD20-CD46 model, several control treatment groups were analyzed, including i.t. injections of purified BiTEs, which had no significant effect on tumor growth and mouse survival on their own, and UV-inactivated viruses, which did not differ significantly in efficacy from non-inactivated counterparts. This result was consistent with observations of low-level measles virus replication in murine tumor cells, indicating that immunogenicity rather than direct oncolysis played the major role in this experimental model. Finally, efficacy of i.t. MV-BiTE was not impaired in MV-immunized animals, suggesting potential of the approach even in measles-seropositive individuals.

Despite the obvious limitations of both in vivo models, this study provided evidence of BiTE-encoding OV efficacy in both an MV-permissive tumor model in immunocompromised mice as well as in a less MV-permissive, immunocompetent model. The syngeneic B16 tumor model is known for low T cell infiltration, which was significantly increased by MV-BiTE treatment. For the first time, endogenous T cells were thus shown to be engaged for in vivo anti-tumor responses by BiTE-encoding OVs. In line with the comparable efficacy of UV-inactivated virus, viral gene expression, replication, and cytotoxicity are likely limited by post-entry restriction factors in the B16 model despite ectopic CD46 expression allowing for viral entry into the tumor cells. The authors speculate that the observed results may therefore translate to even higher efficacy in the human setting with both highly virus-permissive tumors and a complete, though potentially suppressed, endogenous immune system. Correlative immuno-analysis identified T cell exhaustion and inhibition as an obstacle to be addressed in the future, providing a rationale for combinations with further immunotherapeutic approaches, in particular immune checkpoint inhibition, which had been successfully combined with MV treatment before [84, 85].

### Combination of OV-BiTE and CAR T cell therapy to address tumor heterogeneity

While this study demonstrated efficacy of OV-BiTE via recruitment of endogenous T cells, in subsequent studies the OV-BiTE approach was further combined with adoptive transfer of chimeric antigen receptor (CAR) T cells [86]. As an alternative to BiTEs linking the endogenous T cell receptor to a tumor surface antigen, patient-derived T cells can be genetically engineered to express an artificial receptor. Such a CAR provides an extracellular antigen binding domain, a transmembrane domain, and intracellular signaling and co-stimulatory domains for efficient T cell redirection [87]. CAR T cells are activated and expanded ex vivo and reinfused into the patient for cancer therapy. In analogy to the BiTE product Blinatumomab, CD19-specific CAR T cell products have been approved for the treatment of B cell malignancies [88] (see [89, 90] for a recent discussion on the benefits of both approaches). In general, CAR T cell therapy provides a highly active, rapidly expanding effector cell pool but suffers from similar limitations in treating solid tumors as do BiTEs [91] and could also benefit from OV-mediated immunogenic tumor cell lysis [92, 93].

Wing et al. were the first to report combination of OV-BiTE with CAR T cells. By combining ICO15K-cBiTE AdV encoding an EGFR-targeting T cell engager with folate receptor α (FR-α)-specific CAR T cells this group aimed at addressing tumor heterogeneity and potential antigen loss. Indeed, in NSG mice bearing s.c. SKOV3 (human ovarian carcinoma) tumors, the authors showed that selection of FR-α-negative cells occurred upon CAR T cell monotherapy, while EGFR expression was not affected. On-target off-tumor toxicity as a safety concern of redirecting T cells toward tumor-associated antigens via CARs and BiTEs was also addressed in this study. Cetuximab-based EGFR-specific CARs were shown to exert increased cytotoxicity toward healthy keratinocytes and fibroblasts in vitro compared to FR-CARs. This supports the choice of FR-α as the CAR target and OV-mediated, tumor-directed delivery of EGFR-specific BiTEs to avoid systemic exposure.

In vivo efficacy of the combinatorial approach was investigated in two xenograft models in immunodeficient NSG mice, s.c. HCT116 (colorectal cancer) and Panc-1 (pancreatic cancer). Treatment with ICO15K-cBiTE and FR-CARs significantly prolonged survival in the HCT116 model and delayed tumor progression in the Panc-1 model compared to either monotherapy alone. This correlated with enhanced intratumoral CAR T cell accumulation, proliferation, and expression of T cell inhibitory receptors. While both tumor cell lines express high levels of the BiTE target antigen EGFR, expression of the CAR target antigen FR-α differed with intermediate levels in HCT116 and low expression in Panc-1. Interestingly, treatment was efficacious in both models, indicating successful BiTE-mediated re-direction of CAR T cells. In addition, combination of OV-BiTE with FR-CARs was more effective than combination with CD19-specific CAR T cells that do not target a tumor antigen in this setting. This indicates CAR-mediated killing of FR-α-positive tumor cells in addition to BiTE-mediated killing via EGFR targeting. Taken together, this was the first study to show successful combination of OVs, BiTEs, and CARs, suggesting increased efficacy at a tolerable safety profile. Furthermore, this study introduced a strategy to address tumor heterogeneity and antigen loss by concurrent targeting of two distinct tumor surface antigens. However, the immunodeficient NSG model was not well suited to investigate potential synergistic effects including immunogenic oncolysis.

Potentially, CAR T cells in combination with OV-BiTE could benefit from additional immunomodulators such as cytokines and immune checkpoint inhibitors. Recently, another preclinical study tested this hypothesis. Porter et al. combined CAR T cell therapy with an oncolytic helper adenovirus system encoding a BiTE, the T cell-stimulatory cytokine IL-12, and a PD-L1 inhibitor (CAd-Trio) for increased anti-tumor T cell activity [94]. This vector system relies on a replication-competent oncolytic adenovirus and a non-self-replicating helper-dependent packaging virus encoding the immunomodulatory transgenes [95]. The authors previously reported on a variant encoding IL-12p70 and an anti-PD-L1 mini antibody only (CAd-Duo) [96, 97], which was extended here by introducing a sequence encoding a BiTE specific for CD44 variant 6 (CD44v6), a cancer-associated antigen that had been studied as an antibody- and CAR T cell-target before [98, 99].

This construct was tested in two s.c. tumor models in immunodeficient NSG mice, FaDu head and neck squamous cell carcinoma and CAPAN-1 pancreatic adenocarcinoma. CAR T cells targeting HER2 were applied in the FaDu model, and PSCA-specific CAR T cells were used for treatment of CAPAN-1 tumors. In these models, CAd-Trio did not show superior efficacy compared to CAd-Duo. Next, the authors tested the combinatorial approach in an orthotopic model with FaDu cells injected into the mouse tongue. When combined with a suboptimal number of HER2-specific CAR T cells (five times lower than in the s.c. model), CAd-Trio significantly prolonged survival compared to monotherapies. Early anti-tumor efficacy of the CAd-Trio and CAR T cell combination treatment was superior to CAd-Duo plus CAR T cells with regards to tumor reduction and T cell activation. However, this came at the expense of overall and long-term reduced CAR expression and increased PD-L1 levels indicating exhaustion, and the observed survival benefit did not reach statistical significance. The differences in median survival and in early anti-tumor effects between CAd-Duo and CAd-Trio were more pronounced in HER2-deficient FaDu tumors, indicating successful redirection of HER2-specific CAR T cells via the CD44v6-specific BiTE encoded by CAd-Trio. In conclusion, these data indicate efficacy of combined CAR T cell treatment, virus-mediated oncolysis, BiTE-induced T cell redirection and T cell-supporting immunomodulators in an immunodeficient mouse model. The authors now investigate the hypothesis that improved efficacy might be observed in mice harboring a humanized immune system. This study illustrates the impact of model choice on experimental outcome, highlighting the challenge of identifying suitable, i.e., clinically relevant, model systems [100].

### Targeting the tumor microenvironment with OV-BiTE

Rather than targeting tumor cells directly, BiTEs can be designed to direct T cells against pro-tumorigenic components of the tumor microenvironment. This approach was first implemented in OV-BiTE combination therapy using a fibroblast activation protein (FAP)-specific BiTE by Yu et al. [101]. Cancer-associated fibroblasts (CAFs) exert various immunosuppressive and pro-tumorigenic functions, including secretion of transforming growth factor beta (TGF-β), and can be targeted via FAP (reviewed in [102, 103]). As FAP is also expressed on healthy fibroblast subsets during wound repair and tissue remodeling [104, 105], tumor-selective delivery by an OV may avoid toxicities. A Vaccinia-based vector encoding a murine FAP-specific BiTE (mFAP-TEA-VV) was generated analogously to EphA2-TEA-VV described in their earlier study [70]. The latter was also employed here as a control in addition to GM-CSF- and GFP-encoding VVs.

In immunocompetent C57BL/6 mice bearing s.c. B16 tumors, i.t. injections of mFAP-TEA-VV induced a significant reduction in intratumoral FAP-positive cells compared to control viruses. Interestingly, this correlated with increased viral replication, suggesting less hindrance by stromal barriers. Furthermore, mFAP-TEA-VV significantly increased intratumoral T cell infiltration and activation as well as systemic T cell responses against a B16 antigen as evidenced by ex vivo splenocyte ELISpot. Finally, albeit non-significantly, mFAP-TEA-VV delayed tumor progression compared to control viruses, also in uninjected contralateral tumors. mFAP-TEA-VV also led to a reduced number of tumor nodules upon i.v. B16 injection in a lung colonization model. In conclusion, this study provided first proof-of-concept for enhanced viral spread and anti-tumoral T cell responses by targeting tumor-supporting stromal cells with a virus-encoded T cell engager.

This strategy was also investigated using the two previously introduced oncolytic adenovirus platforms. In 2018, Freedman et al. described EnAd-derived vectors encoding a FAP-specific BiTE [106]. A vector encoding a BiTE targeting an irrelevant bacterial antigen was used as a control. As in their previous study, the authors designed vectors with BiTE expression controlled by the constitutively active CMV promoter and the major late promoter via a splice acceptor (SA) site, respectively.

In in vitro co-cultures of normal human dermal fibroblasts with T cells, CMV promoter-driven BiTE expression induced fibroblast killing. For the SA vector, T cell-mediated lysis of FAP-positive cells, also in healthy donor-derived bone marrow samples, required presence of AdV-permissive tumor cells, indicating tumor cell-restricted virus replication and BiTE secretion and thus enhanced safety. Malignant peritoneal ascites samples comprising cancer cells, tumor-associated macrophages (TAMs), CAFs, T cells—a majority of which displayed an exhausted phenotype—and soluble immunosuppressive factors were used as an ex vivo model system to investigate efficacy of EnAd-FAP-BiTE. Cell-free ascites fluid inhibited activation of T cells by anti-CD3/CD28 beads, but not by FAP-BiTEs. Accordingly, in contrast to control vectors, EnAd-FAP-BiTE induced T cell activation and cytotoxicity, leading to depletion of FAP-positive cells and subsequent drops in TGF-β levels, in several patient-derived ascites cultures. These effects were not observed in a patient sample lacking tumor cells, supporting the notion of increased safety by explicit tumor restriction of this vector. Interestingly, not only T cell-related chemokines and effector molecules, but also genes involved in DC maturation and antigen presentation were upregulated upon treatment with EnAd-SA-FAP-BiTE compared to controls in several biopsies. This indicates potential for antigen spread and activation of endogenous, polyclonal T cells to support lasting anti-tumor responses. In addition, EnAd-SA-FAP-BiTE induced repolarization of TAMs from a pro-tumorigenic “M2” toward a more pro-inflammatory “M1” phenotype. Activity of EnAd-SA-FAP-BiTE was furthermore demonstrated on thin tissue slices derived from prostate cancer biopsies. Following virus inoculation in cultivation media, viral infection and BiTE expression were detected in malignant tissue containing EpCAM-positive tumor cells and FAP-expressing CAFs via immunohistochemistry (IHC) and fluorescence microscopy. In contrast to a virus encoding a control BiTE, infection with EnAd-SA-FAP-BiTE resulted in T cell activation and ELISA revealed effector cytokine production. Induction of apoptosis and stromal degradation was restricted to FAP-positive samples as evidenced by IHC, caspase cleavage, and LDH release assays, indicating BiTE-mediated cytotoxicity.

As in their previous publication describing EpCAM-BiTE-encoding vectors, the authors showed efficacy of CAF targeting by EnAd-FAP-BiTE in complementary patient-derived samples ex vivo. This study prompted a clinical trial with an oncolytic adenovirus encoding a FAP-TAc (T cell activator) as well as IFN-α, CXCL9, and CXCL10 to further support T cell activation (PsiOxus T-SIGn platform, NG-641, NCT04053283). The phase Ia/b study will first investigate i.t. or i.v. dose escalations in several epithelial cancer entities and then assess safety and efficacy of the monotherapy compared to combinations with checkpoint inhibition and/or chemotherapy.

CAF-targeting by OV-BiTE was also investigated using the ICOVIR oncolytic adenovirus platform. To this end, ICO15K-FBiTE encoding a FAP-specific BiTE under control of the adenoviral major late promoter was generated, harboring an N-terminal signal peptide and a FLAG-tag at the C-terminus [107]. The two papers by Freedman et al. [106] and de Sostoa et al. [107] describe very similar approaches and constructs and were published only two months apart. In contrast to the EnAd-FAP-BiTE study, de Sostoa et al. employed immunodeficient mouse models instead of clinical samples for efficacy testing.

The mouse models in this study included NSG mice bearing s.c. A549 or HPAC (human pancreatic adenocarcinoma) tumors with i.v. adoptive transfer of human T cells. In both models, i.t. ICO15K-FBiTE treatment delayed tumor growth and significantly prolonged survival compared to control virus or PBS. In the A549 model, this was also associated with higher intratumoral T cell infiltration. High FBiTE expression was detected in ICO15K-FBiTE-treated tumors via RT-qPCR and correlated with significantly decreased mFAP levels compared to PBS-treated tumors in both models. In combination with T cell administration, control virus also prolonged survival and reduced mFAP levels compared to PBS in the A549, but not the HPAC model, suggesting BiTE-independent virus—T cell interactions. Taken together, these findings indicate virus-induced tumor cell lysis and BiTE-dependent, T cell-mediated depletion of mFAP-positive murine stromal cells in vivo upon T cell transfer, resulting in delayed tumor progression and prolonged survival. Efficacy of the OV-FAP-BiTE approach was thus demonstrated in immunocompetent mice [101], in clinical biopsies ex vivo [106], and in immunodeficient mouse models of human tumors [107].

In a recent study, Scott et al. explored OV-encoded T cell-engagers to target another immunosuppressive cell type of the tumor microenvironment, tumor-associated macrophages [108]. As described above, Freedman et al. had previously reported repolarization of M2-like macrophages toward a favorable M1 phenotype by targeting CAFs via EnAd-SA-FAP-BiTE [106]. Here, the authors aimed at TAM depletion via BiTEs targeting the antigens CD206 and folate receptor β (FR-β), respectively. Upregulation of these markers was confirmed on macrophages in the majority of cancer patient-derived ascites samples as well as on healthy monocyte-derived macrophages cultured with ascites fluid.

The authors found that trispecific engagers (TriTEs), harboring an additional CD3 scFv linked to the CD206 BiTE N-terminus, showed increased T cell activation and macrophage killing in co-cultures, also in presence of immunosuppressive ascites fluid. Using malignant ascites-derived cell mixtures, however, both CD206-targeting and non-targeting control TriTE induced similar reduction of CD11b+ CD64+ cells, indicating unspecific toxicity. Interestingly, a different approach to modifying the FR-β BiTE by reversing the scFv order to N-terminal CD3 and C-terminal FR-β targeting increased efficacy of this BiTE in ascites cell mixtures. This variant, termed 3FR as opposed to the original FR3 BiTE, was identified as the most effective also in the context of EnAd-CMV-FR-β vectors. The 3FR BiTE-encoding virus induced stronger T cell activation and CD11b+ CD64+ cell killing in whole ascites samples than empty EnAd, EnAd-CMV-FR3, and viruses encoding control BiTEs. Taken together, Scott et al. demonstrated feasibility of TAM targeting by BiTE-encoding oncolytic adenoviruses in patient-derived malignant ascites models. They devised the addition of further scFvs to generate TriTEs and the optimization of scFv order as two strategies to improve engager molecule function. In addition, a nanobody instead of an scFv was used to target CD206. Nanobodies are derived from heavy chain only antibodies found in camelids [109] and sharks [110] (reviewed in [111]). These findings indicate vast potential of both vector and engager molecule engineering for safer and more efficient cancer immunotherapy, requiring further mechanistic exploration of the OV-BiTE approach.

## Discussion

### OV-BiTE: concept and design

The preclinical studies discussed in this article demonstrate that oncolytic viruses are ideal vehicles for tumor-restricted delivery of BiTEs as immunomodulatory transgenes. In general, transgene design was quite similar across these studies, comprising N-terminal immunoglobulin-derived secretory signal sequences and mostly C-terminal tags for detection purposes. The scFvs were connected via flexible, non-immunogenic glycine-serine linkers. Viral replication kinetics and virus-mediated tumor cell lysis as well as BiTE secretion, binding specificity, and functionality were demonstrated in vitro using appropriate assays. OV-BiTE treatment efficacy in terms of enhanced T cell activation, effector phenotype induction, proliferation, intratumoral infiltration, cytokine production, and cytotoxicity, was demonstrated in complementary tumor models in vitro, in vivo, and ex vivo. These proof-of-concept studies provide strong preclinical evidence for feasibility of the approach for various model target antigens and across distinct vector platforms.

Different classes of potential target antigens exist for cancer therapy. These include (upregulated) tumor-associated antigens like the BiTE and CAR target CD19 that are also found on healthy tissues [1, 112, 113], developmental or cancer-testis antigens whose expression is restricted to certain developmental stages or immune-excluded tissues [114, 115], as well as antigens accessible exclusively on tumor cells, including mutated neoantigens [116] and “liberated” antigens normally present in a bound, closed conformation. The first published OV-BiTE study used such an antigen, EphA2 [70]. Other studies used tumor-associated antigens such as EGFR [73, 74, 86] and EpCAM [77] that are present on vital healthy tissues, leading to toxicities if targeted systemically. Study data show feasibility of OV-mediated, tumor-directed BiTE expression, potentially enabling targeting of such antigens at reduced on-target, off-tumor toxicity. However, safety studies explicitly addressing this promise of OV-BiTE therapy are currently lacking. In contrast to many immunotherapy approaches [117, 118], OV-BiTE directly targets tumor surface antigens and can thus address cancers with immune-evasive defects of the antigen presentation machinery [119]. Of note, OV-BiTE treatment did not induce downregulation of the BiTE target antigen in a mouse model [82].

### Diverse OV platforms in clinical development

Oncolytic vectors from different virus families are currently being tested in clinical studies (see Table 1 for a concise comparison). The majority of all OV-BiTE publications (eight of eleven) investigated adenovirus-based oncolytics, reflecting the current status of clinical virotherapy trials [7]. Adenoviruses are icosahedral DNA viruses which are well-studied, also as non-replicating vectors for gene therapy. Safety concerns have been addressed by genetic modifications restricting virus replication, e.g., to tumor cells with p53 and/or pRb mutations (reviewed in [120]). Adenovirus characteristics and structural properties enable high-titer production and efficient purification. Besides Enadenotucirev (EnAd) (NCT02636036, NCT02028117, NCT02028442, NCT03916510, NCT02053220), which was further engineered to generate the EnAd-EpCAM-BiTE and EnAd-FAP-BiTE vectors described in this review, DNX-2401 is among the clinically most advanced adenovirus-based oncolytics (NCT03896568, NCT03178032, NCT02798406, NCT01956734, NCT02197169, NCT00805376)]. In addition, DNX-2440 is investigated in a Phase I trial (NCT03714334).

Vaccinia vectors were used in two OV-BiTE studies. Vaccinia viruses are DNA viruses derived from the live smallpox vaccine. VV oncolytic properties, safety, and large transgene capacity have been demonstrated in a number of preclinical and clinical studies (reviewed in [121]). JX-594 or PexaVec, a VV encoding GM-CSF, had advanced to a phase III clinical trial for the treatment of hepatocellular carcinoma (NCT02562755), for which enrolment was stopped prematurely without safety concerns following an interim futility analysis [122]. Further studies investigating treatment with different oncolytic Vaccinia viruses, e.g., GL-ONC1 (NCT02759588, NCT01766739) and T601 (NCT04226066), and PexaVec in combination with immune checkpoint inhibitors (NCT03206073, NCT02977156), highlight the potential of this particular oncolytic virus platform for clinical translation.

Oncolytic measles viruses are derived from live attenuated vaccine strains developed against infection with wild-type measles virus, a single-stranded negative sense RNA virus of the *Paramyxoviridae* family known to cause the potentially severe measles disease [123]. Safety and tolerability of these vaccine strains and oncolytic MV have been extensively documented in numerous studies [28]. Oncolytic measles vectors are furthermore characterized by high immunogenicity, natural oncotropism, and flexibility in terms of genetic modification (reviewed in [26, 27, 29, 123, 124]). One bottleneck in the development of oncolytic measles viruses is large-scale production and purification of infectious progeny [125, 126]. The most advanced vectors in clinical trials encode CEA as a soluble biomarker for viral gene expression and human sodium/iodide symporter (NIS), respectively, for imaging and radiotherapy (reviewed in [28]). Remarkably, a durable complete remission was observed in a multiple myeloma patient receiving a single high dose of MV-NIS i.v. [30, 127].

A recent study used a fourth OV, reovirus, for preconditioning prior to BiTE treatment rather than directly using BiTE-encoding vectors [128]. In this paper, reovirus treatment of immunocompetent mice bearing s.c. KPC3 murine pancreatic tumors was shown to induce an interferon response in tumor tissue, followed by an influx of immune effector cells. This resulted in an immune microenvironment conducive to BiTE therapy. In line with these findings, the authors identified sequential treatment with reovirus and BiTE as the most effective treatment order in complementary murine tumor models. However, this approach lacks one of the main advantages of the OV-BiTE strategy, local BiTE expression at the site of viral infection to reduce systemic BiTE-mediated side effects outside of the tumor. Nevertheless, the study illustrates that the precise mechanisms of action and kinetics of immunovirotherapy remain to be fully understood and need to be harnessed for optimal therapeutic efficacy.

As outlined above, the OV-BiTE approach has been successfully implemented with several distinct OV vector platforms, all of which have potential for clinical application. To enable a rational choice of optimal vectors for particular treatment indications and patient subpopulations, systematic comparisons will be required in the future and efforts toward this end have been initiated [129, 130]. However, a major challenge is to identify appropriate models allowing for direct comparison of distinct OVs. Thus, various vector platforms are being developed for cancer immunotherapy, each of which will likely have benefits for particular applications.

One impediment to OV therapy is premature viral clearance, either by hepatic uptake or virus-neutralizing antibodies, depending on the specific OV (reviewed in [131]). One means to circumvent these limitations are carrier cells. This has also been demonstrated for BiTE-encoding OVs by Barlabé et al. [74]. Virus specificity and tumor targeting can be enhanced by further virus modifications, including re-targeting of viral attachment proteins and polymer coating to prevent antibody neutralization (reviewed in [132]).

Moreover, additional possibilities of virus engineering, especially encoding additional transgenes, open a plethora of opportunities to further improve efficacy. Previously described immunomodulatory transgenes encoded by oncolytic vectors include cytokines [21, 133] and immune checkpoint inhibitors [84, 134]. A recent study demonstrated successful combination of several immunomodulators on the same oncolytic adenovirus vector system: In xenograft models with CAR T cell transfer, treatment with viruses encoding a cytokine and a checkpoint inhibitor in addition to a BiTE was superior to viruses lacking the BiTE transgene or encoding the BiTE alone [94].

### Source, specificity and functionality of T cells

Aside from diverse OV vectors, different sources of T cells have been employed in OV-BiTE studies. Efficacy has been demonstrated with endogenous and adoptively transferred, unmodified and genetically engineered T cells in the existing literature. Importantly, endogenous T cells in immunocompetent mouse models and in primary ex vivo samples, having experienced immunosuppressive microenvironments, were shown to be successfully engaged via OV-BiTEs [77, 82, 101, 106, 107]. Despite limitations with regards to displaying more complex immune interactions, adoptive transfer in immunodeficient mouse models allowed for assessment of OV-BiTE efficacy in presence and absence of T cells. Furthermore, genetically engineered T cells can be applied, as shown for combinations of OV-BiTEs with CAR T cells addressing tumor antigen heterogeneity [86, 94]. Ectopic expression of conventional [135] or engineered T cell receptors [136] add to the repertoire of T cell re-direction approaches. One recent strategy utilizes engineered T cells to express BiTEs [137, 138], aiming at redirecting T cells in situ. However, as opposed to OV delivery, this may not necessarily overcome current limitations of BiTE therapy regarding intratumoral infiltration of T cells and on-target off-tumor toxicities. In addition, tumor-infiltrating virus-specific T cells induced by OV therapy can represent a potent effector cell pool for BiTE-mediated tumor cell killing [128]. An alternative approach makes use of a truncated CD19 antigen encoded by an oncolytic Vaccinia virus, aiming at directing CD19-specific CAR T cells toward virus-infected cells [139]. Interestingly, enhanced viral spread was observed upon CAR T cell killing of infected cells. While the study convincingly demonstrates successful preclinical implementation, OV-encoded cell surface antigens lack the advantage of bystander killing of uninfected cells provided by encoding secreted BiTEs.

Irrespective of T cell source and specificity, T cell functionality is decisive for OV-BiTE efficacy. This can be limited by immune checkpoints. Accordingly, different OV platforms have been combined successfully with checkpoint inhibition in preclinical [61, 62, 84, 85, 140] and clinical [60] studies, including the aforementioned BiTE-encoding AdV [94]. However, this is not the only potentially detrimental mechanism of immunosuppresion.

### The complexity of the tumor microenvironment

The multi-faceted interactions between OV-BiTE therapy and T cells are complicated further by the tumor microenvironment (TME). While containing stromal barriers and immunosuppressive factors limiting treatment efficacy, the TME can be deployed to improve therapeutic outcome. For example, not only T cells and OVs, but also macrophages can be genetically modified to secrete BiTEs. In a s.c. glioblastoma xenograft model with i.v. T cell injection in NSG mice, i.t. administration of engineered macrophages secreting EGFRvIII-BiTEs resulted in enhanced T cell responses and delayed tumor progression [141]. When combined with IL-12-secreting macrophages in addition, tumor growth was completely abrogated in this model, indicating the potential of TME modulation. Accordingly, OV-BiTEs targeting components of the tumor microenvironment, namely tumor-associated macrophages and cancer-associated fibroblasts (CAFs), were shown to be successful in several preclinical studies [101, 106, 107]. Recently, an oncolytic adenovirus has been shown to simultaneously target both glioblastoma cells and CAF-like pericytes in a murine brain tumor model [142]. Further approaches harnessing the interplay between virotherapy and the tumor microenvironment have been reviewed elsewhere [103, 143]. These include addressing the tumor vasculature [53, 144], myeloid-derived suppressor cells [145–147], and tumor cell subsets like tumor-initiating [148] or cancer stem cells [149].

The intricate interplay of combinations using advanced oncolytic vectors, T cells, and TME targeting therapeutics results in an infinite complexity, which does not allow systematic testing of all possible combinations. This may be addressed by in silico approaches using experimental data to simulate outcome of hypothetical treatments to prioritize the most promising strategies (reviewed in [150–153]).

### Considerations for clinical translation

Major challenges in taking the OV-BiTE approach forward toward clinical translation are the choice of appropriate BiTE targets, of best-suited OV platforms for particular indications, and, importantly, of appropriate models that optimally reflect the human setting in terms of pharmacology and predicting side effects. The preclinical studies presented in this review have demonstrated proof-of-concept in complementary tumor models including ex vivo samples from human patients and more artificial mouse models that provide three-dimensional organ orientation and interconnection but have limitations regarding virus susceptibility and/or adequate immune responses. In addition, GMP-compatible upscaling of OV-BiTE vector production for clinical testing is not trivial (reviewed in [154]). Generation of viruses in a laboratory for preclinical research differs fundamentally from development and manufacturing of a novel medical product. Regulatory guidelines are complex and need to be addressed from an early development stage on to ensure safe and successful clinical translation (reviewed in [155]). Producer cell and virus seed batches, cultivating and harvesting procedures, downstream purification and identity validation have to be pre-defined and constantly controlled for quality. Although oncolytic vector platforms differ in their respective production processes, standardized protocols enable GMP-compatible manufacturing. For some viruses, e.g., vesicular stomatitis virus, high-titer production of purified progeny can be relatively fast and straightforward [156]. Others, such as measles virus-derived oncolytics, are harder to produce at high titers and purification is aggravated by a pleomorphic structure and sensitivity to pH changes and shear stress [125, 126, 157]. Combinations with advanced immunotherapeutics such as CAR T cells require further consideration in terms of target, design, scalability, safety, and the regulatory framework (reviewed in [158]).

Although these considerations prevent rapid translation of most approaches, the first clinical OV-BiTE trial has already been initiated: NG-641, an EnAd-derived vector armed with a FAP-specific BiTE (FAP-TAc antibody) and an additional immune-stimulatory molecule (CXCL9-CXCL10-IFNa), is being investigated both as i.v. and i.t. treatment across different epithelial tumor entities (NCT04053283). Study completion is expected for the end of 2022. The results are eagerly awaited and may provide further insights into safety/tolerability and efficacy of immunotherapy with BiTE-encoding oncolytic viruses and potentially beneficial combination regimens.

The OV-BiTE approach is one of several immunovirotherapeutic strategies now entering clinical investigation. GM-CSF encoding derivatives of several OV platforms have been studied in numerous clinical trials (reviewed in [56]). These include the clinically most advanced oncolytic therapeutics: T-VEC, derived from a herpes simplex virus type I and approved for treatment of malignant melanoma following a successful study [21], and vaccinia-based PexaVec, which had progressed to a phase III trial in hepatocellular carcinoma (NCT02562755). In addition, OVs encoding a range of novel transgenes are now in Phase I/II studies. These include cytokines and T cell costimulatory molecules, with candidates such as TILT-123, an oncolytic adenovirus encoding TNF-α and IL-2 (NCT04217473) and delolimogene mupadenorepvec/LOAd703 encoding membrane-bound CD40L and 4-1BBL (NCT04123470, NCT02705196, NCT03225989). As in LOAd703, combinations of two or multiple immunomodulators are not uncommon. Examples include RIVAL 01/TBio-6517, a Vaccinia virus for tumor-directed expression of FLT3L, anti-CTLA-4, and IL-12 (NCT04301011), and ONCR-177 (NCT04348916), an HSV-1 mediating expression of IL-12, FLT3LG, CCL4, anti-PD-1, and anti-CTLA-4 to enhance induction of tumor-specific adaptive immunity. Another variation of the oncolytic vaccination paradigm is to encode tumor antigens within the viral vector as a heterologous cancer vaccine. Studies with MG-1 Maraba viruses encoding HPV E6/E7 and MAGEA3, respectively, are ongoing (NCT03618953, NCT03773744, NCT02879760). In the majority of trials, these viral vectors are combined with systemic PD-1 immune checkpoint blockade. The design of these trials illustrates the significant advancement and increasing complexity of the field. These developments underline the necessity of both preclinical and translational research as drivers of progress in modern medical oncology. Complementary research even in early-stage clinical development can provide first-hand insight to decipher which approaches will bring most benefit for cancer patients. Identifying correlates of response and resistance will aid in prioritizing and refining treatment strategies in immunovirotherapy.

## Conclusions

In this regard, the OV-BiTE approach is one example. Starting from a clear scientific rationale, several independent preclinical studies have provided proof-of-concept for this strategy. Thus, OV-BiTEs must now demonstrate applicability and efficacy in clinical reality. Variations of vector platform and target molecules as well as combination therapies offer a plethora of opportunities for improvement. Therefore, early clinical investigations should also address mechanistic aspects to provide avenues for further development.

## Data Availability

Not applicable.

## Abbreviations

AdV: Oncolytic adenovirus
APCs: Antigen-presenting cells
BiTEs: Bispecific T cell engagers
CAd: Combinatorial adenoviral vector system
CAFs: Cancer-associated fibroblasts
CAR: Chimeric antigen receptor
CD44v6: CD44 variant 6
CEA: Carcinoembryonic antigen
CMV: Cytomegalovirus
CTLA-4: Cytotoxic T lymphocyte-associated protein 4,
CTx: Chemotherapy
DAMPs and PAMPs: Danger- and pathogen-associated molecular patterns
ds: Double-stranded
EGFR: Epidermal growth factor receptor
EnAd: Enadenotucirev
enc.: Encoding
EpCAM: Epithelial cell adhesion molecule
EphA2: Ephrin type 2 receptor
FAP: Fibroblast activation protein
FHA: *B. pertussis* Filamentous hemagglutinin adhesin
Flt3L: Flt3 ligand
FR-α: Folate receptor α
FR-β: Folate receptor β
GBM: Glioblastoma multiforme
GM-CSF: Granulocyte macrophage colony-stimulating factor
GMP: Good manufacturing practice
GS: Gliosarcoma
H-1PV: H-1 protoparvovirus
HCC: Hepatocellular carcinoma
HNSCC: Head and neck squamous cell carcinoma
HVEM: Herpes virus entry mediator
i.p.: Intraperitoneally
i.t.: Intratumoral
i.v.: Intravenous
ICAM: Intercellular adhesion molecule
ICD: Immunogenic tumor cell death
ICI: Immune checkpoint inhibition
IHC: Immunohistochemistry
IL-12: Interleukin-12
ITx: Immunotherapy
JAM-A: Junctional adhesion molecule A
LDLR: Low-density lipoprotein receptor
MAGE: Melanoma antigen
MenSCs: Mesenchymal stem cells derived from menstrual blood
MV: Measles virus
NIS: Sodium/iodide symporter
NMIBC: Non-muscle invasive bladder cancer
NSCLC: Non-small cell lung cancer
OV-BiTE: BiTE-encoding oncolytic virus
OVs: Oncolytic viruses
PBMCs: Peripheral blood mononuclear cells
PD-L1: Programmed death-ligand 1
PVRL: Poliovirus-receptor-like
RTx: Radiotherapy
s.c.: Subcutaneously
SA: Splice acceptor
SCC: Squamous cell carcinoma
scFv: Single-chain variable fragments
ss: Single-stranded
TAA: Tumor-associated antigen
TAc: T cell activator
TAMs: Tumor-associated macrophages
TGF-β: Transforming growth factor beta
TME: Tumor microenvironment
TriTEs: Trispecific engagers
TTx: Targeted therapy
T-VEC: Talimogene laherparepvec
V_H_ V_L_: Antibody variable heavy and light chains
VV: Vaccinia virus

## References

[1] Yu J, Wang W, Huang H (2019). Efficacy and safety of bispecific T-cell engager (BiTE) antibody blinatumomab for the treatment of relapsed/refractory acute lymphoblastic leukemia and non-Hodgkin's lymphoma: a systemic review and meta-analysis. Hematology (Amsterdam, Netherlands).

[2] Yu S, Li A, Liu Q, Yuan X, Xu H, Jiao D, Pestell RG, Han X, Wu K (2017). Recent advances of bispecific antibodies in solid tumors. J Hematol Oncol.

[3] Goebeler ME, Bargou RC (2020). T cell-engaging therapies-BiTEs and beyond. Nat Rev Clin Oncol.

[4] Esfahani K, Roudaia L, Buhlaiga N, Del Rincon SV, Papneja N, Miller WH (2020). A review of cancer immunotherapy: from the past, to the present, to the future. Curr Oncol.

[5] Waldman AD, Fritz JM, Lenardo MJ (2020). A guide to cancer immunotherapy: from T cell basic science to clinical practice. Nat Rev Immunol.

[6] Cook M, Chauhan A (2020). Clinical application of oncolytic viruses: a systematic review. Int J Mol Sci.

[7] Macedo N, Miller DM, Haq R, Kaufman HL (2020). Clinical landscape of oncolytic virus research in 2020. J Immunother Cancer.

[8] Cao GD, He XB, Sun Q, Chen S, Wan K, Xu X, Feng X, Li PP, Chen B, Xiong MM (2020). The oncolytic virus in cancer diagnosis and treatment. Front Oncol.

[9] Farrera-Sal M, Moya-Borrego L, Bazan-Peregrino M, Alemany R. Evolving status of clinical immunotherapy with oncolytic adenovirus. Clin Cancer Res Off J Am Assoc Cancer Res. 2021. 10.1158/1078-0432.CCR-20-1565 33526422

[10] Hemminki O, Dos Santos JM, Hemminki A (2020). Oncolytic viruses for cancer immunotherapy. J Hematol Oncol.

[11] Peter M, Kuhnel F (2020). Oncolytic adenovirus in cancer immunotherapy. Cancers.

[12] Ruano D, Lopez-Martin JA, Moreno L, Lassaletta A, Bautista F, Andion M, Hernandez C, Gonzalez-Murillo A, Melen G, Alemany R (2020). First-in-human, first-in-child trial of autologous MSCs carrying the oncolytic virus Icovir-5 in patients with advanced tumors. Mol Therapy J Am Soc Gene Therapy.

[13] Machiels JP, Salazar R, Rottey S, Duran I, Dirix L, Geboes K, Wilkinson-Blanc C, Pover G, Alvis S, Champion B (2019). A phase 1 dose escalation study of the oncolytic adenovirus enadenotucirev, administered intravenously to patients with epithelial solid tumors (EVOLVE). J Immunother Cancer.

[14] Small EJ, Carducci MA, Burke JM, Rodriguez R, Fong L, van Ummersen L, Yu DC, Aimi J, Ando D, Working P (2006). A phase I trial of intravenous CG7870, a replication-selective, prostate-specific antigen-targeted oncolytic adenovirus, for the treatment of hormone-refractory, metastatic prostate cancer. Mol Therapy J Am Soc Gene Therapy.

[15] Sin J, Mangale V, Thienphrapa W, Gottlieb RA, Feuer R (2015). Recent progress in understanding coxsackievirus replication, dissemination, and pathogenesis. Virology.

[16] Bradley S, Jakes AD, Harrington K, Pandha H, Melcher A, Errington-Mais F (2014). Applications of coxsackievirus A21 in oncology. Oncolytic Virotherapy.

[17] McCarthy C, Jayawardena N, Burga LN, Bostina M (2019). Developing picornaviruses for cancer therapy. Cancers.

[18] Koch MS, Lawler SE, Chiocca EA (2020). HSV-1 oncolytic viruses from bench to bedside: an overview of current clinical trials. Cancers.

[19] Menotti L, Avitabile E (2020). Herpes simplex virus oncolytic immunovirotherapy: the blossoming branch of multimodal therapy. Int J Mol Sci.

[20] Xu X, Zhang Y, Li Q (2019). Characteristics of herpes simplex virus infection and pathogenesis suggest a strategy for vaccine development. Rev Med Virol.

[21] Andtbacka RH, Kaufman HL, Collichio F, Amatruda T, Senzer N, Chesney J, Delman KA, Spitler LE, Puzanov I, Agarwala SS (2015). Talimogene laherparepvec improves durable response rate in patients with advanced melanoma. J Clin Oncol Off J Am Soc Clin Oncol.

[22] Breitbach CJ (2020). Considerations for clinical translation of MG1 Maraba virus. Methods Mol Biol.

[23] Pol JG, Atherton MJ, Bridle BW, Stephenson KB, Le Boeuf F, Hummel JL, Martin CG, Pomoransky J, Breitbach CJ, Diallo JS (2018). Development and applications of oncolytic Maraba virus vaccines. Oncolytic Virotherapy.

[24] Zemp F, Rajwani J, Mahoney DJ (2018). Rhabdoviruses as vaccine platforms for infectious disease and cancer. Biotechnol Genet Eng Rev.

[25] Engeland CE, Ungerechts G (2021). Measles virus as an oncolytic immunotherapy. Cancers.

[26] Pidelaserra-Marti G, Engeland CE (2020). Mechanisms of measles virus oncolytic immunotherapy. Cytokine Growth Factor Rev.

[27] Leber MF, Neault S, Jirovec E, Barkley R, Said A, Bell JC, Ungerechts G (2020). Engineering and combining oncolytic measles virus for cancer therapy. Cytokine Growth Factor Rev.

[28] Msaouel P, Opyrchal M, Dispenzieri A, Peng KW, Federspiel MJ, Russell SJ, Galanis E (2018). Clinical trials with oncolytic measles virus: current status and future prospects. Curr Cancer Drug Targets.

[29] Aref S, Bailey K, Fielding A (2016). Measles to the rescue: a review of oncolytic measles virus. Viruses.

[30] Packiriswamy N, Upreti D, Zhou Y, Khan R, Miller A, Diaz RM, Rooney CM, Dispenzieri A, Peng KW, Russell SJ (2020). Oncolytic measles virus therapy enhances tumor antigen-specific T-cell responses in patients with multiple myeloma. Leukemia.

[31] Angelova A, Ferreira T, Bretscher C, Rommelaere J, Marchini A (2021). Parvovirus-based combinatorial immunotherapy: a reinforced therapeutic strategy against poor-prognosis solid cancers. Cancers.

[32] Hartley A, Kavishwar G, Salvato I, Marchini A (2020). A roadmap for the success of oncolytic parvovirus-based anticancer therapies. Annu Rev Virol.

[33] Bretscher C, Marchini A (2019). H-1 parvovirus as a cancer-killing agent: past, present, and future. Viruses.

[34] Ferreira T, Kulkarni A, Bretscher C, Richter K, Ehrlich M, Marchini A (2020). Oncolytic H-1 parvovirus enters cancer cells through clathrin-mediated endocytosis. Viruses.

[35] Geletneky K, Huesing J, Rommelaere J, Schlehofer JR, Leuchs B, Dahm M, Krebs O, von Knebel DM, Huber B, Hajda J (2012). Phase I/IIa study of intratumoral/intracerebral or intravenous/intracerebral administration of Parvovirus H-1 (ParvOryx) in patients with progressive primary or recurrent glioblastoma multiforme: ParvOryx01 protocol. BMC Cancer.

[36] Geletneky K, Hajda J, Angelova AL, Leuchs B, Capper D, Bartsch AJ, Neumann JO, Schoning T, Husing J, Beelte B (2017). Oncolytic H-1 parvovirus shows safety and signs of immunogenic activity in a first phase I/IIa glioblastoma trial. Mol Ther J Am Soc Gene Ther.

[37] Hajda J, Lehmann M, Krebs O, Kieser M, Geletneky K, Jager D, Dahm M, Huber B, Schoning T, Sedlaczek O (2017). A non-controlled, single arm, open label, phase II study of intravenous and intratumoral administration of ParvOryx in patients with metastatic, inoperable pancreatic cancer: ParvOryx02 protocol. BMC Cancer.

[38] Gromeier M, Nair SK (2018). Recombinant poliovirus for cancer immunotherapy. Annu Rev Med.

[39] Desjardins A, Gromeier M, Herndon JE, Beaubier N, Bolognesi DP, Friedman AH, Friedman HS, McSherry F, Muscat AM, Nair S (2018). Recurrent glioblastoma treated with recombinant poliovirus. N Engl J Med.

[40] McNamara A, Roebke K, Danthi P. Cell killing by reovirus: mechanisms and consequences. Curr Top Microbiol Immunol. 2020. 10.1007/82_2020_225 PMC11890104 32986138

[41] Muller L, Berkeley R, Barr T, Ilett E, Errington-Mais F (2020). Past, present and future of oncolytic reovirus. Cancers.

[42] Zhang Z, Dong L, Zhao C, Zheng P, Zhang X, Xu J (2021). Vaccinia virus-based vector against infectious diseases and tumors. Hum Vaccin Immunother.

[43] Pelin A, Boulton S, Tamming LA, Bell JC, Singaravelu R (2020). Engineering vaccinia virus as an immunotherapeutic battleship to overcome tumor heterogeneity. Expert Opin Biol Ther.

[44] Torres-Dominguez LE, McFadden G (2019). Poxvirus oncolytic virotherapy. Expert Opin Biol Ther.

[45] Kretzschmar M, Wallinga J, Teunis P, Xing S, Mikolajczyk R (2006). Frequency of adverse events after vaccination with different vaccinia strains. PLoS Med.

[46] Isaacs SN (2019). Working safely with vaccinia virus: laboratory technique and review of published cases of accidental laboratory infections with poxviruses. Methods Mol Biol.

[47] Silva NIO, de Oliveira JS, Kroon EG, Trindade GS, Drumond BP (2020). Here, there, and everywhere: the wide host range and geographic distribution of zoonotic orthopoxviruses. Viruses.

[48] Miest TS, Cattaneo R (2014). New viruses for cancer therapy: meeting clinical needs. Nat Rev Microbiol.

[49] Seymour LW, Fisher KD (2016). Oncolytic viruses: finally delivering. Br J Cancer.

[50] Russell SJ, Peng KW, Bell JC (2012). Oncolytic virotherapy. Nat Biotechnol.

[51] Ilkow CS, Marguerie M, Batenchuk C, Mayer J, Ben Neriah D, Cousineau S, Falls T, Jennings VA, Boileau M, Bellamy D (2015). Reciprocal cellular cross-talk within the tumor microenvironment promotes oncolytic virus activity. Nat Med.

[52] Arulanandam R, Batenchuk C, Angarita FA, Ottolino-Perry K, Cousineau S, Mottashed A, Burgess E, Falls TJ, De Silva N, Tsang J (2015). VEGF-mediated induction of PRD1-BF1/Blimp1 expression sensitizes tumor vasculature to oncolytic virus infection. Cancer Cell.

[53] Breitbach CJ, De Silva NS, Falls TJ, Aladl U, Evgin L, Paterson J, Sun YY, Roy DG, Rintoul JL, Daneshmand M (2011). Targeting tumor vasculature with an oncolytic virus. Mol Ther J Am Soc Gene Ther.

[54] Lichty BD, Breitbach CJ, Stojdl DF, Bell JC (2014). Going viral with cancer immunotherapy. Nat Rev Cancer.

[55] Achard C, Surendran A, Wedge ME, Ungerechts G, Bell J, Ilkow CS (2018). Lighting a fire in the tumor microenvironment using oncolytic immunotherapy. EBioMedicine.

[56] Pol JG, Workenhe ST, Konda P, Gujar S, Kroemer G. Cytokines in oncolytic virotherapy. Cytokine Growth Factor Rev. 2020. 10.1016/j.cytogfr.2020.10.007 33183957

[57] Twumasi-Boateng K, Pettigrew JL, Kwok YYE, Bell JC, Nelson BH (2018). Oncolytic viruses as engineering platforms for combination immunotherapy. Nat Rev Cancer.

[58] Oh CM, Chon HJ, Kim C (2020). Combination immunotherapy using oncolytic virus for the treatment of advanced solid tumors. Int J Mol Sci.

[59] Shi T, Song X, Wang Y, Liu F, Wei J (2020). Combining oncolytic viruses with cancer immunotherapy: establishing a new generation of cancer treatment. Front Immunol.

[60] Ribas A, Dummer R, Puzanov I, VanderWalde A, Andtbacka RHI, Michielin O, Olszanski AJ, Malvehy J, Cebon J, Fernandez E (2017). Oncolytic virotherapy promotes intratumoral T cell infiltration and improves anti-PD-1 immunotherapy. Cell.

[61] Samson A, Scott KJ, Taggart D, West EJ, Wilson E, Nuovo GJ, Thomson S, Corns R, Mathew RK, Fuller MJ (2018). Intravenous delivery of oncolytic reovirus to brain tumor patients immunologically primes for subsequent checkpoint blockade. Sci Transl Med.

[62] Bourgeois-Daigneault MC, Roy DG, Aitken AS, El Sayes N, Martin NT, Varette O, Falls T, St-Germain LE, Pelin A, Lichty BD (2018). Neoadjuvant oncolytic virotherapy before surgery sensitizes triple-negative breast cancer to immune checkpoint therapy. Sci Transl Med.

[63] Haitz K, Khosravi H, Lin JY, Menge T, Nambudiri VE (2020). Review of talimogene laherparepvec: a first-in-class oncolytic viral treatment of advanced melanoma. J Am Acad Dermatol.

[64] van Akkooi ACJ, Haferkamp S, Papa S, Franke V, Pinter A, Weishaupt C, Huber MA, Loquai C, Richtig E, Gokani P (2021). A retrospective chart review study of real-world use of talimogene laherparepvec in unresectable stage IIIB-IVM1a melanoma in four European countries. Adv Ther.

[65] Galanis E, Atherton PJ, Maurer MJ, Knutson KL, Dowdy SC, Cliby WA, Haluska P, Long HJ, Oberg A, Aderca I (2015). Oncolytic measles virus expressing the sodium iodide symporter to treat drug-resistant ovarian cancer. Can Res.

[66] Larocca CA, LeBoeuf NR, Silk AW, Kaufman HL (2020). An update on the role of talimogene laherparepvec (T-VEC) in the treatment of melanoma: best practices and future directions. Am J Clin Dermatol.

[67] Song X-T (2013). Combination of virotherapy and T-cell therapy: arming oncolytic virus with T-cell engagers. Discov Med.

[68] Scott EM, Duffy MR, Freedman JD, Fisher KD, Seymour LW (2018). Solid tumor immunotherapy with T cell engager-armed oncolytic viruses. Macromol Biosci.

[69] Guo ZS, Lotze MT, Zhu Z, Storkus WJ, Song XT (2020). Bi- and tri-specific T cell engager-armed oncolytic viruses: next-generation cancer immunotherapy. Biomedicines.

[70] Yu F, Wang X, Guo ZS, Bartlett DL, Gottschalk SM, Song XT (2014). T-cell engager-armed oncolytic vaccinia virus significantly enhances antitumor therapy. Mol Ther J Am Soc Gene Ther.

[71] Coffman KT, Hu M, Carles-Kinch K, Tice D, Donacki N, Munyon K, Kifle G, Woods R, Langermann S, Kiener PA (2003). Differential EphA2 epitope display on normal versus malignant cells. Can Res.

[72] Albelda SM, Thorne SH (2014). Giving oncolytic vaccinia virus more BiTE. Mol Ther J Am Soc Gene Ther.

[73] Fajardo CA, Guedan S, Rojas LA, Moreno R, Arias-Badia M, de Sostoa J, June CH, Alemany R (2017). Oncolytic adenoviral delivery of an EGFR-targeting T-cell engager improves antitumor efficacy. Can Res.

[74] Barlabé P, Sostoa J, Fajardo CA, Alemany R, Moreno R (2020). Enhanced antitumor efficacy of an oncolytic adenovirus armed with an EGFR-targeted BiTE using menstrual blood-derived mesenchymal stem cells as carriers. Cancer Gene Ther.

[75] Moreno R, Fajardo CA, Farrera-Sal M, Perisé-Barrios AJ, Morales-Molina A, Al-Zaher AA, García-Castro J, Alemany R (2019). Enhanced antitumor efficacy of oncolytic adenovirus-loaded menstrual blood-derived mesenchymal stem cells in combination with peripheral blood mononuclear cells. Mol Cancer Ther.

[76] Moreno R, Rojas LA, Villellas FV, Soriano VC, García-Castro J, Fajardo CA, Alemany R (2017). Human menstrual blood-derived mesenchymal stem cells as potential cell carriers for oncolytic adenovirus. Stem Cells Int.

[77] Freedman JD, Hagel J, Scott EM, Psallidas I, Gupta A, Spiers L, Miller P, Kanellakis N (2017). Oncolytic adenovirus expressing bispecific antibody targets T-cell cytotoxicity in cancer biopsies. EMBO Mol Med.

[78] Gires O, Pan M, Schinke H, Canis M, Baeuerle PA (2020). Expression and function of epithelial cell adhesion molecule EpCAM: where are we after 40 years?. Cancer Metastasis Rev.

[79] Scott EM, Frost S, Khalique H, Freedman JD, Seymour LW, Lei-Rossmann J (2020). Use of liquid patient ascites fluids as a preclinical model for oncolytic virus activity. Methods Mol Biol.

[80] Garcia-Carbonero R, Salazar R, Duran I, Osman-Garcia I, Paz-Ares L, Bozada JM, Boni V, Blanc C, Seymour L, Beadle J (2017). Phase 1 study of intravenous administration of the chimeric adenovirus enadenotucirev in patients undergoing primary tumor resection. J Immunother Cancer.

[81] Illingworth S, Di Y, Bauzon M, Lei J, Duffy MR, Alvis S, Champion B, Lieber A, Hermiston T, Seymour LW (2017). Preclinical safety studies of enadenotucirev, a chimeric group B human-specific oncolytic adenovirus. Mol Ther Oncolytics.

[82] Speck T, Heidbuechel JPW, Veinalde R, Jaeger D, von Kalle C, Ball CR, Ungerechts G, Engeland CE (2018). Targeted BiTE expression by an oncolytic vector augments therapeutic efficacy against solid tumors. Clin Cancer Res Off J Am Assoc Cancer Res.

[83] Heidbuechel JPW, Engeland CE (2019). Paramyxoviruses for tumor-targeted immunomodulation: design and evaluation ex vivo. JoVE.

[84] Engeland CE, Grossardt C, Veinalde R, Bossow S, Lutz D, Kaufmann JK, Shevchenko I, Umansky V, Nettelbeck DM, Weichert W (2014). CTLA-4 and PD-L1 checkpoint blockade enhances oncolytic measles virus therapy. Mol Ther J Am Soc Gene Ther.

[85] Hardcastle J, Mills L, Malo CS, Jin F, Kurokawa C, Geekiyanage H, Schroeder M, Sarkaria J, Johnson AJ, Galanis E (2017). Immunovirotherapy with measles virus strains in combination with anti-PD-1 antibody blockade enhances antitumor activity in glioblastoma treatment. Neuro Oncol.

[86] Wing A, Fajardo CA, Posey AD, Shaw C, Da T, Young RM, Alemany R, June CH, Guedan S (2018). Improving CART-cell therapy of solid tumors with oncolytic virus-driven production of a bispecific T-cell engager. Cancer Immunol Res.

[87] June CH, Sadelain M (2018). Chimeric antigen receptor therapy. N Engl J Med.

[88] Frigault MJ, Maus MV (2020). State of the art in CAR T cell therapy for CD19+ B cell malignancies. J Clin Investig.

[89] Subklewe M (2021). BiTEs better than CAR T cells. Blood Adv.

[90] Molina JC, Shah NN (2021). CAR T cells better than BiTEs. Blood Adv.

[91] Slaney CY, Wang P, Darcy PK, Kershaw MH (2018). CARs versus BiTEs: a comparison between T cell-redirection strategies for cancer treatment. Cancer Discov.

[92] Ajina A, Maher J (2017). Prospects for combined use of oncolytic viruses and CAR T-cells. J Immunother Cancer.

[93] Guedan S, Alemany R (2018). CAR-T cells and oncolytic viruses: joining forces to overcome the solid tumor challenge. Front Immunol.

[94] Porter CE, Rosewell Shaw A, Jung Y, Yip T, Castro PD, Sandulache VC, Sikora A, Gottschalk S, Ittman MM, Brenner MK (2020). Oncolytic adenovirus armed with BiTE, Cytokine, and checkpoint inhibitor enables CAR T cells to control the growth of heterogeneous tumors. Mol Ther J Am Soc Gene Ther.

[95] Farzad L, Cerullo V, Yagyu S, Bertin T, Hemminki A, Rooney C, Lee B, Suzuki M (2014). Combinatorial treatment with oncolytic adenovirus and helper-dependent adenovirus augments adenoviral cancer gene therapy. Mol Ther Oncolytics.

[96] Rosewell Shaw A, Porter CE, Watanabe N, Tanoue K, Sikora A, Gottschalk S, Brenner MK, Suzuki M (2017). Adenovirotherapy delivering cytokine and checkpoint inhibitor augments CAR T cells against metastatic head and neck cancer. Mol Ther J Am Soc Gene Ther.

[97] Tanoue K, Rosewell Shaw A, Watanabe N, Porter C, Rana B, Gottschalk S, Brenner M, Suzuki M (2017). Armed oncolytic adenovirus-expressing PD-L1 mini-body enhances antitumor effects of chimeric antigen receptor T cells in solid tumors. Can Res.

[98] Rupp U, Schoendorf-Holland E, Eichbaum M, Schuetz F, Lauschner I, Schmidt P, Staab A, Hanft G, Huober J, Sinn HP (2007). Safety and pharmacokinetics of bivatuzumab mertansine in patients with CD44v6-positive metastatic breast cancer: final results of a phase I study. Anticancer Drugs.

[99] Casucci M, Nicolis di Robilant B, Falcone L, Camisa B, Norelli M, Genovese P, Gentner B, Gullotta F, Ponzoni M, Bernardi M (2013). CD44v6-targeted T cells mediate potent antitumor effects against acute myeloid leukemia and multiple myeloma. Blood.

[100] Hegde PS, Chen DS (2020). Top 10 challenges in cancer immunotherapy. Immunity.

[101] Yu F, Hong B, Song X-T (2017). A T-cell engager-armed oncolytic vaccinia virus to target the tumor stroma. Cancer Transl Med.

[102] Chen X, Song E (2019). Turning foes to friends: targeting cancer-associated fibroblasts. Nat Rev Drug Discov.

[103] Everts A, Bergeman M, McFadden G, Kemp V (2020). Simultaneous tumor and stroma targeting by oncolytic viruses. Biomedicines.

[104] Levy MT, McCaughan GW, Abbott CA, Park JE, Cunningham AM, Müller E, Rettig WJ, Gorrell MD (1999). Fibroblast activation protein: a cell surface dipeptidyl peptidase and gelatinase expressed by stellate cells at the tissue remodelling interface in human cirrhosis. Hepatology (Baltimore, MD).

[105] Jacob M, Chang L, Puré E (2012). Fibroblast activation protein in remodeling tissues. Curr Mol Med.

[106] Freedman JD, Duffy MR, Lei-Rossmann J, Muntzer A, Scott EM, Hagel J, Campo L, Bryant RJ, Verrill C, Lambert A (2018). An oncolytic virus expressing a T-cell engager simultaneously targets cancer and immunosuppressive stromal cells. Cancer Res.

[107] de Sostoa J, Fajardo CA, Moreno R, Ramos MD, Farrera-Sal M, Alemany R (2019). Targeting the tumor stroma with an oncolytic adenovirus secreting a fibroblast activation protein-targeted bispecific T-cell engager. J Immunother Cancer.

[108] Scott EM, Jacobus EJ, Lyons B, Frost S, Freedman JD, Dyer A, Khalique H, Taverner WK, Carr A, Champion BR (2019). Bi- and tri-valent T cell engagers deplete tumour-associated macrophages in cancer patient samples. J Immunother Cancer.

[109] Hamers-Casterman C, Atarhouch T, Muyldermans S, Robinson G, Hamers C, Songa EB, Bendahman N, Hamers R (1993). Naturally occurring antibodies devoid of light chains. Nature.

[110] Greenberg AS, Avila D, Hughes M, Hughes A, McKinney EC, Flajnik MF (1995). A new antigen receptor gene family that undergoes rearrangement and extensive somatic diversification in sharks. Nature.

[111] Muyldermans S (2013). Nanobodies: natural single-domain antibodies. Annu Rev Biochem.

[112] Wang K, Wei G, Liu D (2012). CD19: a biomarker for B cell development, lymphoma diagnosis and therapy. Exp Hematol Oncol.

[113] Neelapu SS (2019). Managing the toxicities of CAR T-cell therapy. Hematol Oncol.

[114] Salmaninejad A, Zamani MR, Pourvahedi M, Golchehre Z, Hosseini Bereshneh A, Rezaei N (2016). Cancer/testis antigens: expression, regulation, tumor invasion, and use in immunotherapy of cancers. Immunol Invest.

[115] Gordeeva O (2018). Cancer-testis antigens: unique cancer stem cell biomarkers and targets for cancer therapy. Semin Cancer Biol.

[116] Schumacher TN, Scheper W, Kvistborg P (2019). Cancer neoantigens. Annu Rev Immunol.

[117] Haen SP, Löffler MW, Rammensee HG, Brossart P (2020). Towards new horizons: characterization, classification and implications of the tumour antigenic repertoire. Nat Rev Clin Oncol.

[118] Vigneron N (2015). Human tumor antigens and cancer immunotherapy. Biomed Res Int.

[119] Fisher K, Hazini A, Seymour LW (2021). Tackling HLA deficiencies head on with oncolytic viruses. Cancers.

[120] Baker AT, Aguirre-Hernández C, Halldén G, Parker AL (2018). Designer oncolytic adenovirus: coming of age. Cancers.

[121] Guo ZS, Lu B, Guo Z, Giehl E, Feist M, Dai E, Liu W, Storkus WJ, He Y, Liu Z (2019). Vaccinia virus-mediated cancer immunotherapy: cancer vaccines and oncolytics. J Immunother Cancer.

[122] Transgene provides update on PHOCUS study of Pexa-Vec in liver cancer following planned interim futility analysis. https://www.businesswire.com/news/home/20190802005141/en/Transgene-Provides-Update-on-PHOCUS-Study-of-Pexa-Vec-in-Liver-Cancer-Following-Planned-Interim-Futility-Analysis.

[123] Russell SJ, Peng KW (2009). Measles virus for cancer therapy. Curr Top Microbiol Immunol.

[124] Mühlebach MD (2020). Measles virus in cancer therapy. Curr Opin Virol.

[125] Langfield KK, Walker HJ, Gregory LC, Federspiel MJ (2011). Manufacture of measles viruses. Methods Mol Biol.

[126] Loewe D, Dieken H, Grein TA, Weidner T, Salzig D, Czermak P (2020). Opportunities to debottleneck the downstream processing of the oncolytic measles virus. Crit Rev Biotechnol.

[127] Russell SJ, Federspiel MJ, Peng KW, Tong C, Dingli D, Morice WG, Lowe V, O'Connor MK, Kyle RA, Leung N (2014). Remission of disseminated cancer after systemic oncolytic virotherapy. Mayo Clin Proc.

[128] Groeneveldt C, Kinderman P, van den Wollenberg DJM, van den Oever RL, Middelburg J, Mustafa DAM, Hoeben RC, van der Burg SH, van Hall T, van Montfoort N (2020). Preconditioning of the tumor microenvironment with oncolytic reovirus converts CD3-bispecific antibody treatment into effective immunotherapy. J Immunother Cancer.

[129] Kemp V, Lamfers MLM, van der Pluijm G, van den Hoogen BG, Hoeben RC (2020). Developing oncolytic viruses for clinical use: a consortium approach. Cytokine Growth Factor Rev.

[130] Cervera-Carrascon V, Quixabeira DCA, Havunen R, Santos JM, Kutvonen E, Clubb JHA, Siurala M, Heiniö C, Zafar S, Koivula T (2020). Comparison of clinically relevant oncolytic virus platforms for enhancing T cell therapy of solid tumors. Mol Ther Oncolytics.

[131] Ferguson MS, Lemoine NR, Wang Y (2012). Systemic delivery of oncolytic viruses: hopes and hurdles. Adv Virol.

[132] Hill C, Carlisle R (2019). Achieving systemic delivery of oncolytic viruses. Expert Opin Drug Deliv.

[133] Liu BL, Robinson M, Han ZQ, Branston RH, English C, Reay P, McGrath Y, Thomas SK, Thornton M, Bullock P (2003). ICP34.5 deleted herpes simplex virus with enhanced oncolytic, immune stimulating, and anti-tumour properties. Gene Ther.

[134] Dias JD, Hemminki O, Diaconu I, Hirvinen M, Bonetti A, Guse K, Escutenaire S, Kanerva A, Pesonen S, Loskog A (2012). Targeted cancer immunotherapy with oncolytic adenovirus coding for a fully human monoclonal antibody specific for CTLA-4. Gene Ther.

[135] Robbins PF, Kassim SH, Tran TL, Crystal JS, Morgan RA, Feldman SA, Yang JC, Dudley ME, Wunderlich JR, Sherry RM (2015). A pilot trial using lymphocytes genetically engineered with an NY-ESO-1-reactive T-cell receptor: long-term follow-up and correlates with response. Clin Cancer Res Off J Am Assoc Cancer Res.

[136] Baeuerle PA, Ding J, Patel E, Thorausch N, Horton H, Gierut J, Scarfo I, Choudhary R, Kiner O, Krishnamurthy J (2019). Synthetic TRuC receptors engaging the complete T cell receptor for potent anti-tumor response. Nat Commun.

[137] Iwahori K, Kakarla S, Velasquez MP, Yu F, Yi Z, Gerken C, Song XT, Gottschalk S (2015). Engager T cells: a new class of antigen-specific T cells that redirect bystander T cells. Mol Ther J Am Soc Gene Ther.

[138] Choi BD, Yu X, Castano AP, Bouffard AA, Schmidts A, Larson RC, Bailey SR, Boroughs AC, Frigault MJ, Leick MB (2019). CAR-T cells secreting BiTEs circumvent antigen escape without detectable toxicity. Nat Biotechnol.

[139] Park AK, Fong Y, Kim SI, Yang J, Murad JP, Lu J, Jeang B, Chang WC, Chen NG, Thomas SH (2020). Effective combination immunotherapy using oncolytic viruses to deliver CAR targets to solid tumors. Sci Transl Med.

[140] Zamarin D, Holmgaard RB, Subudhi SK, Park JS, Mansour M, Palese P, Merghoub T, Wolchok JD, Allison JP (2014). Localized oncolytic virotherapy overcomes systemic tumor resistance to immune checkpoint blockade immunotherapy. Sci Transl Med.

[141] Gardell JL, Matsumoto LR, Chinn H, DeGolier KR, Kreuser SA, Prieskorn B, Balcaitis S, Davis A, Ellenbogen RG, Crane CA (2020). Human macrophages engineered to secrete a bispecific T cell engager support antigen-dependent T cell responses to glioblastoma. J Immunother Cancer.

[142] Li M, Li G, Kiyokawa J, Tirmizi Z, Richardson LG, Ning J, Das S, Martuza RL, Stemmer-Rachamimov A, Rabkin SD (2020). Characterization and oncolytic virus targeting of FAP-expressing tumor-associated pericytes in glioblastoma. Acta Neuropathol Commun.

[143] Jamieson TR, Poutou J, Ilkow CS (2020). Redirecting oncolytic viruses: engineering opportunists to take control of the tumour microenvironment. Cytokine Growth Factor Rev.

[144] Matuszewska K, Santry LA, van Vloten JP, AuYeung AWK, Major PP, Lawler J, Wootton SK, Bridle BW, Petrik J (2019). Combining vascular normalization with an oncolytic virus enhances immunotherapy in a preclinical model of advanced-stage ovarian cancer. Clin Cancer Res Off J Am Assoc Cancer Res.

[145] Meng G, Li B, Chen A, Zheng M, Xu T, Zhang H, Dong J, Wu J, Yu D, Wei J (2020). Targeting aerobic glycolysis by dichloroacetate improves Newcastle disease virus-mediated viro-immunotherapy in hepatocellular carcinoma. Br J Cancer.

[146] Katayama Y, Tachibana M, Kurisu N, Oya Y, Terasawa Y, Goda H, Kobiyama K, Ishii KJ, Akira S, Mizuguchi H (2018). Oncolytic reovirus inhibits immunosuppressive activity of myeloid-derived suppressor cells in a TLR3-dependent manner. J Immunol (Baltimore, Md: 1950).

[147] Ding AS, Routkevitch D, Jackson C, Lim M (2019). Targeting myeloid cells in combination treatments for glioma and other tumors. Front Immunol.

[148] Bach P, Abel T, Hoffmann C, Gal Z, Braun G, Voelker I, Ball CR, Johnston IC, Lauer UM, Herold-Mende C (2013). Specific elimination of CD133+ tumor cells with targeted oncolytic measles virus. Can Res.

[149] Crupi MJF, Bell JC, Singaravelu R (2019). Concise review: targeting cancer stem cells and their supporting niche using oncolytic viruses. Stem Cells.

[150] Santiago DN, Heidbuechel JPW, Kandell WM, Walker R, Djeu J, Engeland CE, Abate-Daga D, Enderling H (2017). Fighting cancer with mathematics and viruses. Viruses.

[151] Heidbuechel JPW, Abate-Daga D, Engeland CE, Enderling H (2020). Mathematical modeling of oncolytic virotherapy. Methods Mol Biol.

[152] Wodarz D (2016). Computational modeling approaches to the dynamics of oncolytic viruses. Wiley Interdiscip Rev Syst Biol Med.

[153] Wodarz D, Hofacre A, Lau JW, Sun Z, Fan H, Komarova NL (2012). Complex spatial dynamics of oncolytic viruses in vitro: mathematical and experimental approaches. PLoS Comput Biol.

[154] Ungerechts G, Bossow S, Leuchs B, Holm PS, Rommelaere J, Coffey M, Coffin R, Bell J, Nettelbeck DM (2016). Moving oncolytic viruses into the clinic: clinical-grade production, purification, and characterization of diverse oncolytic viruses. Mol Therapy Methods Clin Dev.

[155] Barkholt L, Voltz-Girolt C, Raine J, Salmonson T, Schüssler-Lenz M (2019). Regulatory watch: European regulatory experience with advanced therapy medicinal products. Nat Rev Drug Discovery.

[156] Ausubel LJ, Meseck M, Derecho I, Lopez P, Knoblauch C, McMahon R, Anderson J, Dunphy N, Quezada V, Khan R (2011). Current good manufacturing practice production of an oncolytic recombinant vesicular stomatitis viral vector for cancer treatment. Hum Gene Ther.

[157] Grein TA, Loewe D, Dieken H, Weidner T, Salzig D, Czermak P (2019). Aeration and shear stress are critical process parameters for the production of oncolytic measles virus. Front Bioeng Biotechnol.

[158] Hartmann J, Schussler-Lenz M, Bondanza A, Buchholz CJ (2017). Clinical development of CAR T cells-challenges and opportunities in translating innovative treatment concepts. EMBO Mol Med.

